# Mathematical modelling of vaccination rollout and NPIs lifting on COVID-19 transmission with VOC: a case study in Toronto, Canada

**DOI:** 10.1186/s12889-022-13597-9

**Published:** 2022-07-15

**Authors:** Elena Aruffo, Pei Yuan, Yi Tan, Evgenia Gatov, Iain Moyles, Jacques Bélair, James Watmough, Sarah Collier, Julien Arino, Huaiping Zhu

**Affiliations:** 1 Centre for Disease Modelling (CDM), York University, 4700 Keele Street, Toronto, ON M3J1P3 Canada; 2 Department of Mathematics and Statistics, York University, 4700 Keele Street, Toronto, ON M3J1P3 Canada; 3 Toronto Public Health, City of Toronto, Toronto, ON Canada; 4 Département de Mathématiques Et de Statistique, Université de Montréal, Montréal, Québec Canada; 5 Department of Mathematics and Statistics, University of New Brunswick, Fredericton, New Brunswick Canada; 6 Department of Mathematics, University of Manitoba, Winnipeg, MB Canada

**Keywords:** COVID-19, SARS-CoV-2, Mathematical modeling, Age structure, Nonpharmaceutical Interventions, Vaccine, Waning, Resurgence, VOC

## Abstract

**Background:**

Since December 2020, public health agencies have implemented a variety of vaccination strategies to curb the spread of SARS-CoV-2, along with pre-existing Nonpharmaceutical Interventions (NPIs). Initial strategies focused on vaccinating the elderly to prevent hospitalizations and deaths, but with vaccines becoming available to the broader population, it became important to determine the optimal strategy to enable the safe lifting of NPIs while avoiding virus resurgence.

**Methods:**

We extended the classic deterministic SIR compartmental disease-transmission model to simulate the lifting of NPIs under different vaccine rollout scenarios. Using case and vaccination data from Toronto, Canada between December 28, 2020, and May 19, 2021, we estimated transmission throughout past stages of NPI escalation/relaxation to compare the impact of lifting NPIs on different dates on cases, hospitalizations, and deaths, given varying degrees of vaccine coverages by 20-year age groups, accounting for waning immunity.

**Results:**

We found that, once coverage among the elderly is high enough (80% with at least one dose), the main age groups to target are 20–39 and 40–59 years, wherein first-dose coverage of at least 70% by mid-June 2021 is needed to minimize the possibility of resurgence if NPIs are to be lifted in the summer. While a resurgence was observed for every scenario of NPI lifting, we also found that under an optimistic vaccination coverage (70% coverage by mid-June, along with postponing reopening from August 2021 to September 2021) can reduce case counts and severe outcomes by roughly 57% by December 31, 2021.

**Conclusions:**

Our results suggest that focusing the vaccination strategy on the working-age population can curb the spread of SARS-CoV-2. However, even with high vaccination coverage in adults, increasing contacts and easing protective personal behaviours is not advisable since a resurgence is expected to occur, especially with an earlier reopening.

**Supplementary Information:**

The online version contains supplementary material available at 10.1186/s12889-022-13597-9.

## Background

Prior to December 2020, implementation of nonpharmaceutical interventions (NPIs), including school/business closures, physical distancing, and mask-wearing, was the main tool to control the spread of SARS-CoV-2. However, with the development of effective vaccines against SARS-CoV-2, in December 2020 many countries were able to initiate vaccination campaigns [1–3]. The most recommended strategy was prioritizing the elderly and high-risk populations, followed by essential workers, and then the general public [4–6]. At the initial stage of vaccine distribution, strict NPIs were kept in place to avoid potential virus resurgence. After almost six months of immunization, the focus shifted in establishing an optimal vaccination strategy in order to safely lift NPIs while avoiding virus resurgence.

There have been numerous mathematical models aiming to identify the best vaccination strategy [7–16]. Earlier models focused on reducing the disease spread and identifying priority groups for receiving their first doses. For example, Matrajt et al. [16] developed a deterministic model with age structure to determine which age group should be vaccinated first. They showed that with low coverage, the elderly (60 + years) must be prioritized to reduce the number of deaths. Bubar et al. [9] showed that prioritizing younger ages (20–49 years) can reduce cumulative cases independently from rollout speeds and coverages. In Fall 2020, new, more transmissible, and virulent, variants of concern (VOCs) were discovered [17–20]. In many areas of the world, VOCs cases increased rapidly in the following months and the VOCs became dominant over SARS-CoV-2 wildtype [20–23]. Giordano et al. [8] investigated the impact of mass vaccination campaigns and NPI lifting while considering increased transmission due to VOCs. They found that NPIs implementation is crucial even after the rollout of a vaccine. Moore et al. [11] also introduced VOCs in their age-structured model as well as vaccination and different levels of reopening. They similarly confirmed that relaxing NPIs too early will result in virus resurgence and noted the infeasibility of reaching herd immunity through vaccination. While vaccination of young children was approved much later, some previous studies still considered younger age groups when defining the best vaccine rollout for minimizing infections, hospitalizations, and deaths [9, 24, 25]. Meehan et al. [24], for example, showed that prioritizing individuals aged 30–59 reduces the transmission, while prioritizing ages 65 + reduces deaths. They also found that when coverage is close to levels required for herd immunity, vaccinating middle-aged adults should be prioritized, since vaccinated young teenagers and children appear to have minimal impact. Shim et al. [25] conducted an optimization analysis on vaccination strategies, considering age groups and vaccine efficacy. They found that for a vaccine with at least 70% efficacy, targeting ages 20–49 is best for reducing infections, while targeting ages 50 + is best for reducing mortality. Although existing studies provide important information for decision making, they do not capture the nuances around the impact of VOCs on vaccination efforts by age group in terms of both transmission and virulence, as well as vaccine effectiveness, in order to assess scenarios for the safe lifting of NPIs at various timepoints.

In this paper, we aimed to determine an optimal vaccination strategy to enable the safe lifting of NPIs while avoiding virus resurgence, using Toronto, Canada as a case study. We have extended the basic SIR compartmental model to reflect a variety of infectious and recovered states and incorporated age structure and vaccine status. We further included two strains of the virus, differentially affecting transmission, virulence, and vaccine effectiveness. We then assessed different reopening strategies given varying degrees of vaccine coverage by age group aiming to reduce infections, hospitalizations, and deaths.

## Methods

### Data, model structure and assumptions

Our model is applicable to any geographical region where sufficiently detailed data are available. To study COVID-19 vaccination rollout and reopening strategies, we used data from Toronto, Canada between December 28, 2020 and May 19, 2021. To calibrate model parameters, we used data on cases, deaths, hospitalizations, and daily vaccine doses, publicly available at the City of Toronto website [26]. Parameters were estimated by Least Square Method (LSM), minimizing the sum of square of data and model’s predictions to find the best fit. To address the uncertainty in the process of parameter estimation, we conduct 500 replications while the parameter sets’ initial guesses were sampled by the Latin Hypercube Sampling method with normal distribution. Afterward, we evaluated the mean value, standard deviation, and confidence interval of the collection of best-fits where the parameters lie. Further details including the fit and confidence intervals for parameters are shown in Supplementary Information (SI), Figure SI2.

**Fig. 1:**
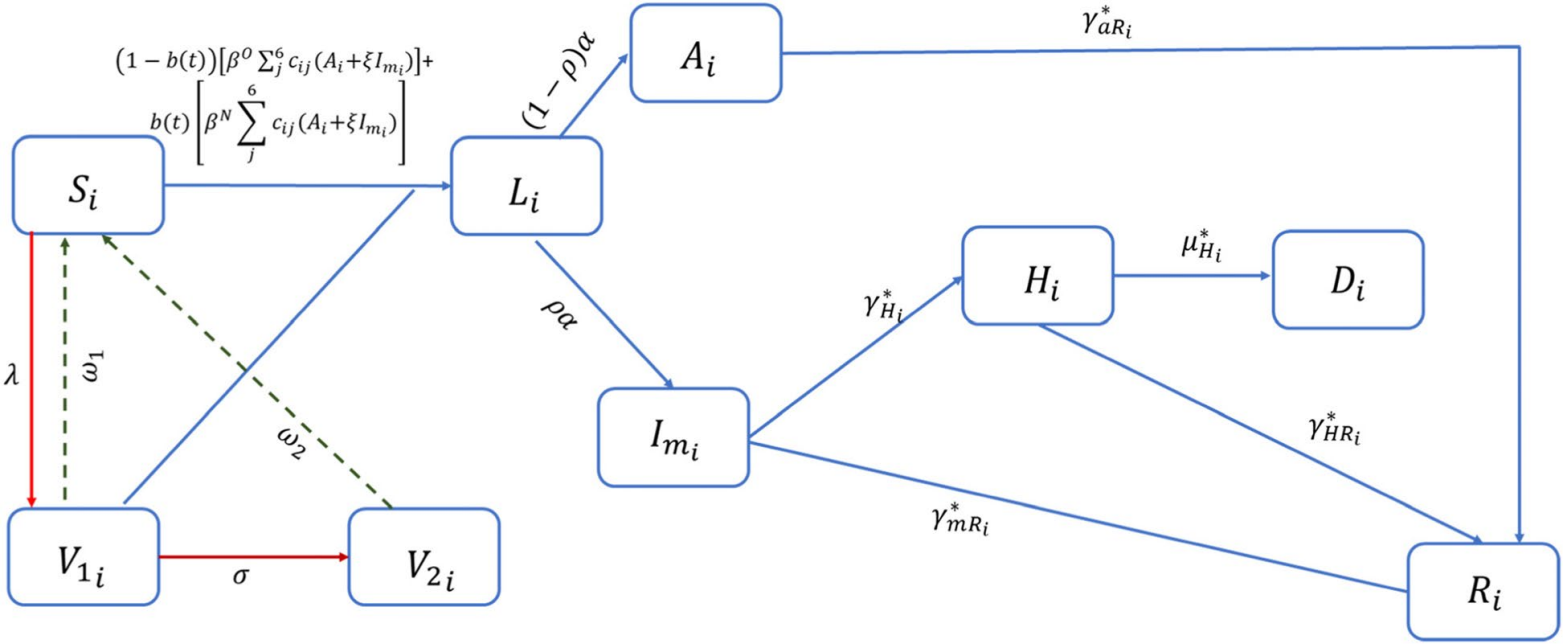
Flow diagram of COVID-19 transmission dynamics with two vaccination processes. Acronyms: i ∈ {1–6}Age groups: 0–9 (unvaccinated), 10–19, 20–39, 40–59, 60–79, 80+ ; In Age group *i*: S_i_ (Susceptible), L_i_ (Latently infected), A_i_ (Asymptomatic infected), I_mi_ (Symptomatic mild infected), H_i_ (Hospitalized), D_i_ (Deceased), R_i_ (Recovered), V_1i_ (Vaccinated with first dose), V_2i_ (Vaccinated with second dose). To capture the different infection severities coming from VOC or wildtype variant, each disease-state progression is variant-dependent (* = wildtype or VOC). Red arrows: vaccination process. Dashed lines: waning process. Model assumptions: • Only susceptible individuals, aged 10+ years, will receive the vaccine. Vaccine reduces susceptibility. Partially vaccinated people can become infected and infectious if the vaccine is not efficient. • Immunity follows two steps: partial (receiving 1 dose) and full (receiving 2 doses), with the second dose given after 112 days (in some predictive scenarios after 50 or 21 days). Immunity from one dose wanes in 120 days and from two doses after 365 days. Vaccination continues until 80% of the entire population receive at least one dose. • Vaccine efficacy is age-dependent (higher for teenagers and adults, 10% lower for elderly) and is the same against wildtype variant and VOC (all non-wildtype cases are assumed to be B.1.1.7 variant). • VOC and wildtype are both included in the transmission process, assuming that the proportion of VOC cases increases over time following a sigmoidal function, with transmission from VOC 1.5 times higher than wildtype. • Only individuals hospitalized might die from the infection. ${\beta }^{O}, {\beta }^{N}$: • probability of transmission; ${c}_{ij}$: contacts rate between individuals in age group i and individuals in age group j; $\xi$: proportion of infectious individuals not respecting isolation; $\lambda$: daily vaccine doses; ${\omega }_{1}, {\omega }_{2}$: waning rates, after one or two doses; $\sigma$: average time between doses; $\rho$: proportion of individuals developing symptoms; ${\gamma }_{H}$: hospitalization rate; ${\mu }_{H}$: death rate; ${\gamma }_{aR}, {\gamma }_{mR}, {\gamma }_{HR}$: recovery rate of asymptomatic, infectious and hospitalized individuals

As of May 5, 2021, the Canadian government approved the use of the Pfizer-BioNTech COVID-19 vaccine in teenagers aged 12 + years [27]. We divided the population into 6 age groups: 0–9, 10–19, 20–39, 40–59, 60–79, 80 + years. Vaccination for children 5–11 was approved in Canada in November 2021. As our study ended in May 2021, 0–9 years were not considered for vaccination. However, they are included for transmission consideration. We extended the SIR disease states to further include latent, asymptomatic, and symptomatic infections, as well as population movement into hospitalization, recovery, or death states (i.e., SLAIHDR model, Fig. 1). We incorporated two strains of the virus: wildtype and VOC (specifically, the B.1.1.7 (Alpha) variant, most commonly circulating at the time of model parametrization).

The infection dynamic is presented in Fig. 1. The susceptible compartment (S), with age-dependent susceptibility, can become infected with either the wildtype or VOC (indicated with O and N, respectively), with age-dependent transmission rate$\beta {c}_{ij}$, where ${c}_{ij}$ is the number of contacts of individuals in age group *i* with individuals in age groups j and $\beta$ is the probability of infection per contact. Multiple studies confirmed that the Alpha VOC is between 40 and 90% more transmissible than the wildtype variant [28–30], hence we assumed that the probability of transmission of VOC (${\beta }^{N})$ is $\zeta$ times higher than the probability of transmission of the wildtype (i.e.,${\beta }^{N}=\zeta {\beta }^{o}$). Upon infection, individuals enter a latent stage (compartment L) where they are neither symptomatic nor infectious. We assume latently infected individuals become infectious at rate α: a fraction $\rho$ develops mild symptoms (compartment ${I}_{m}$) and the remainder remain asymptomatic for the duration of infection (compartment A). From A, individuals recover at rate ${\gamma }_{aR}$. We further assume individuals with mild symptoms recover, at rate${\gamma }_{mR}$, or progress to clinical stage (compartment H), at rate${\gamma }_{H}$, and reduce their contact rate. In H, individuals can either recover, at rate${\gamma }_{HR}$, or become deceased (D) at rate ${\mu }_{H}$. To better describe the daily increase of cases resulting from the VOC, we modelled the growth of cases from the novel variant using a sigmoidal function (Figure SI1). Each compartment is divided into $1-b(t)$ proportion coming from the wildtype variant and $b\left(t\right)$ proportion coming from the VOC. This allowed us to capture the differences between these variants in terms of probabilities of transmission and probabilities of severe outcomes. The model’s equations and parameters are shown in Eq. SI1 and Table SI2.

The population is further structured by vaccination status (none, partial and full), with no possibility of reinfection. The vaccine we chose to model has the characteristics of Pfizer/Moderna in that it is delivered in two doses [31]. Therefore, individuals move to ${V}_{1}$ after receiving the first dose and ${V}_{2}$ after receiving the second dose, where they are considered as fully immunized. We assumed that eventually all recipients of the first dose will be vaccinated with the second one and this occurs at a rate $\sigma$. Vaccine efficacy, $\epsilon$, of both doses is included and assumed the same against VOC and wildtype variants. We assumed that vaccine efficacy reduces the probability of infection ($\beta {c}_{ij}$) by $1-\epsilon$. Immunity induced by vaccination is assumed to wane after one and two doses at different rates. In the model, we simulated a minimum vaccine coverage that each age group needs to reach by June 14, 2021. Thereafter, the vaccination process continues until 80% of the population is vaccinated. We assumed this maximum coverage based on the reopening strategies (depending on the vaccination coverage) implemented by the Government of Ontario [32].

### Reopening scenarios analysis

With increasing vaccination rollout, public health has considered easing some NPI restrictions [32]. Therefore, we predicted cumulative cases and deaths, and daily hospitalizations until December 31, 2021, comparing different degrees of reopening, at different dates, given a variety of vaccine coverages for each age group. Using historical case data and information on previous policy periods of escalating/de-escalating NPIs in Toronto, we have identified four distinct stages of transmission with varying degrees to which indoor/outdoor gatherings were permitted: retail at full, limited, or curbside-only capacity, and whether indoor/outdoor dining and other sectors, including cinemas and gyms, were open. Compared to the level of restriction in Toronto up to May 19, 2021, during which retail was curbside only, with no indoor/outdoor dining, stay-at-home in effect, and personal protective (PP) behaviours such as physical distancing and mask-wearing enforced, we included possible permutations for reopening on June 15, August 15, or September 15 to different degrees (1) No reopening (i.e., remain at baseline); (2) Partial reopening, whereby contacts are increased by 50% compared to baseline, reflecting a small increase in gatherings and retail; (3) Total reopening with contacts increased by 70% and the probability of transmission increasing by 35%, compared to baseline, reflecting most sectors being open with a more relaxed use of PP behaviour; and (4) Pre-pandemic contact rates with no limitations on gatherings and with all sectors being open, while still maintaining PP behaviour. The number of pre-pandemic contacts within and between age groups were calculated using contact matrices from Canada [33]. Matrices are provided as overall mixing and for specific locations (such as work and school). For our study, we employed the overall mixing matrix and aggregated some age groups from this matrix to match the age groups used in our model (for detailed calculations and matrix, see Table SI3). For each permutation, we calculated the effective reproduction number ${R}_{c}$ using the Next Generation Matrix method [34, 35], and identified the vaccination coverage, by age, that would be required to reduce ${R}_{c}$ below 1.

For each reopening scenario, we examined the impact of vaccination by age group. We used vaccination data up to May 19 to estimate the vaccination rate required to reach specific coverages by June 14, 2021 (a plateau, or a 10%, 20% or 30% increase from current coverage for each age group), all the model permutations are given in Figure SI3. We then used the average daily doses from that day moving forward, until 80% of the population has received at least one dose. Since the vaccine coverage by May among ages 60 + was above 70%, we primarily focus on varying coverages in those under 60 years of age, assuming that by mid-June, ages 60–79 and 80 + might reach 80%-90% coverage with the first dose, ages 40–50 might reach 70%-90%, ages 20–39 might reach 60%-80%, and ages 10–19 might reach 20%-40%. Given guidelines on extended timeframes with limited vaccine supply [36], we assumed that the second dose is given 112 days later, also compared scenarios with a shorter interval between doses (50 or 21 days). The first and second dose are assumed to be 80% and 90% effective, respectively, with 10% reduction in effectiveness among ages 80 + (see Table SI2).

### Sensitivity analysis

To explore the impact that vaccine- and infection- related parameters have on the model outcomes, we conducted a sensitivity analysis using the Latin Hypercube Sampling-Partial Rank Correlation Coefficient (LHS-PRCC) method. We generated 1000 samples using the LHS method with uniform distribution and investigated correlation between the samples and model outputs, such as cumulative cases and deaths. The scenario used is total reopening in September. PRCC values above 0 indicate that the parameter is positively correlated to the outcome, indicating that as the parameter value increases, the outcome increases. Conversely, PRCC values below 0 indicate that the parameter and the output are negatively correlated, indicating that as the parameter value increases, the outcome decreases (and vice versa). Parameters with an absolute PRCC value greater than 0.5 are considered significant [37, 38]. Ranges of sampled parameters are shown in Table SI2.

### Uncertainty of the parameters

To further investigate the uncertainty of the model parameters, we projected the hospitalizations under partial reopening using the parameters in the confidence interval. We compared the scenario with highest and lowest coverages (Figure SI4). We observe that with lowest coverages, hospitalizations are much higher than those with maximum coverages in all age groups. We can also observe that within the confidence interval, the mean value (solid lines) follows the trend of the confidence interval, hence we use the mean values to generate our projections.

## Results

### Effective reproduction number

We investigated the effective reproduction number ($${R}_{c})$$ considering that the coverage of the age groups 10–19, 60–79 and 80 + years is 20%, 80%, 90%, respectively, and varying the coverages for the remaining groups from 50 to 90%. The susceptible compartment is reduced daily by a time-depended vaccination rate, reflecting the daily doses given to each age group. The effective reproduction number depends on the proportion of individuals vaccinated by age and the level of NPIs restrictions and PP behaviours, each of which in our model are time dependent. Hence, we investigated the effective reproduction number based on the total coverage by age group that might be achieved.

We observe that, with high level of restrictions, the reproduction number remains below 1 if at least 50% of adults aged 20- 59 are vaccinated (see Figure SI5A). If current contacts are increased by 50% and PP behaviors are in place, the reproduction number remains below 1 if a minimum coverage of 90% is achieved in both the age groups 20–39 and 40–59 years (see SI Figure SI5B). On the other hand, if total reopening or pre-pandemic reopening occurs, at any time, the reproduction number will be above 1, with highest values for pre-pandemic reopening (see Figure SI5C-D).

### Prediction of optimal reopening strategies and vaccination coverages

The figures in the following sections represent the percentage change of the shown scenario with respect to the baseline case, defined as the minimum coverages of each sub-populations and no reopening scenario (see Figure SI3).

#### Age group targets for minimizing cases, deaths and hospitalizations

**Fig. 2:**
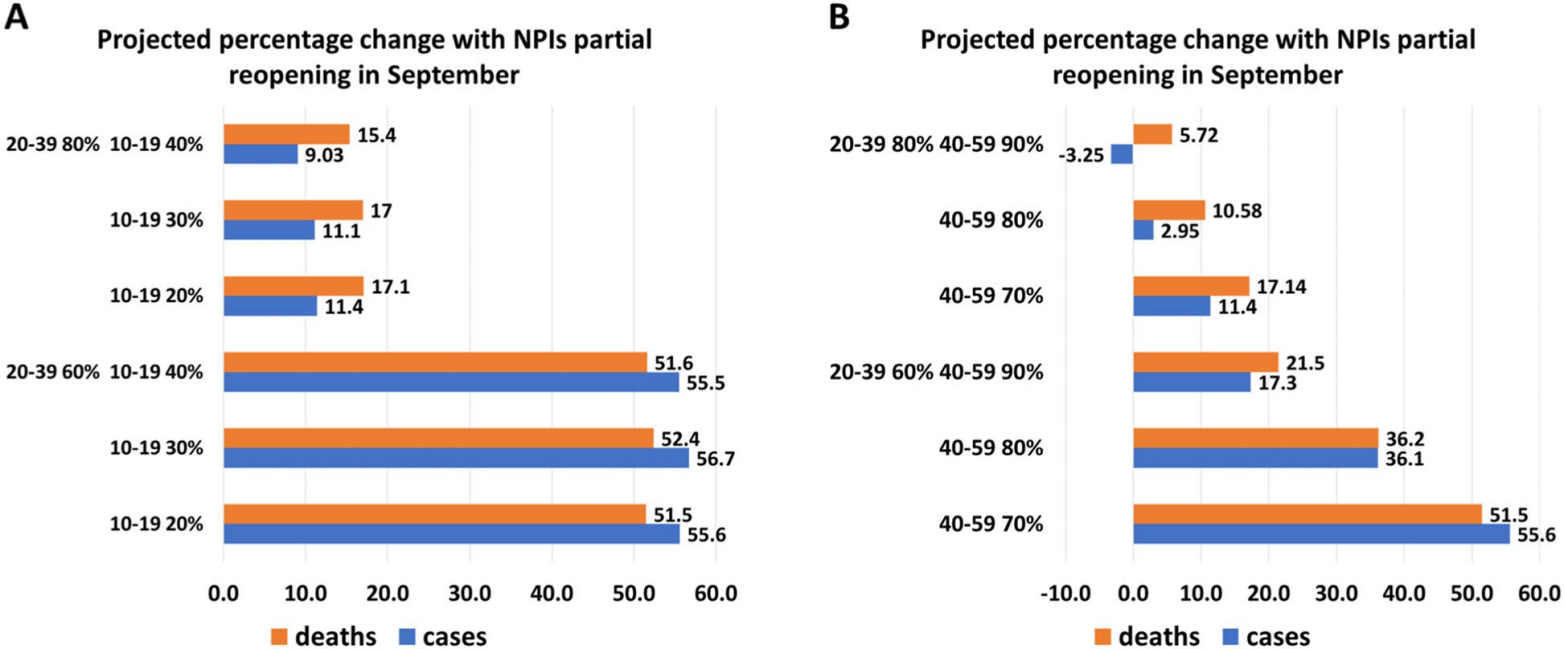
Percentage change of cumulative cases and deaths with respect to the baseline no reopening in SI Figure SI3 with partial reopening in September, when age groups 60–79 and 80 + reach coverages 80%, 90% by June 14. Cases and deaths are reported comparing different coverages for age group 10–19 years, assuming 40–59 years fixed at 70% coverage and comparing different coverages for age group 40–59 years, assuming 10–19 years fixed at 20% coverage. The second dose is given at a rate of 1/112 days.^−1^. We observe that the highest increase occurs when the sub-populations’ coverage is the minimum level considered. Moreover, keeping adults aged between 50–69 years at 70% and increase the coverages of teenagers does not provide significant reduction in cases nor deaths. On the other hand, the smallest increase is provided by the highest coverage in the two adults age groups

Figure 2 shows the percentage change of cumulative cases and deaths by the end of December 2021, with respect to the baseline in Figure SI3, when partial reopening occurs on September 15. In general, when efforts are mainly put into vaccinating the 10–19 years age group (Fig. 2A), the change in cases does not appear to be significant, unless individuals in the 20–39 years age group reach a higher coverage. For example, with a 60% coverage of the 20–39 age group, if vaccine coverage in the youngest group is increased from 20 to 40%, cases are reduced from 55.6% to 55.5%. If the youngest age group is at minimum coverage (i.e., 20%), the cumulative cases remarkably decrease as the coverage of age groups 20–39 years and 40–59 years increase (Fig. 2B). We observe that if 20–39- and 40–59-years groups are vaccinated above 80%, the increase from the baseline varies between 2.95% and -3.25%. If the 20–39 age group reaches 80% coverage, then increasing the coverage of the 40–59 age group from 70 to 80% or 90% reduces cases by 74% and 128%, respectively.

We note that the increase with 30% coverage among teenagers is slightly higher than the ones with 20% coverage. This result is because after June 14 the vaccination process continues until the total eligible population reaches 80%. If we increase the vaccination rate of the 10–19 age group, the total coverage is reached earlier leaving some age groups still susceptible. In particular, the age group 40–59 years will not reach sufficient coverage to prevent the increase of cases.

The results for cumulative deaths are similar to those for cumulative cases. Since the elderly population is already highly vaccinated, it is important to focus on the immunization of the age groups 20–39 and 40–59 years reach at least 80% coverage to reduce deaths after reopening.

**Fig. 3:**
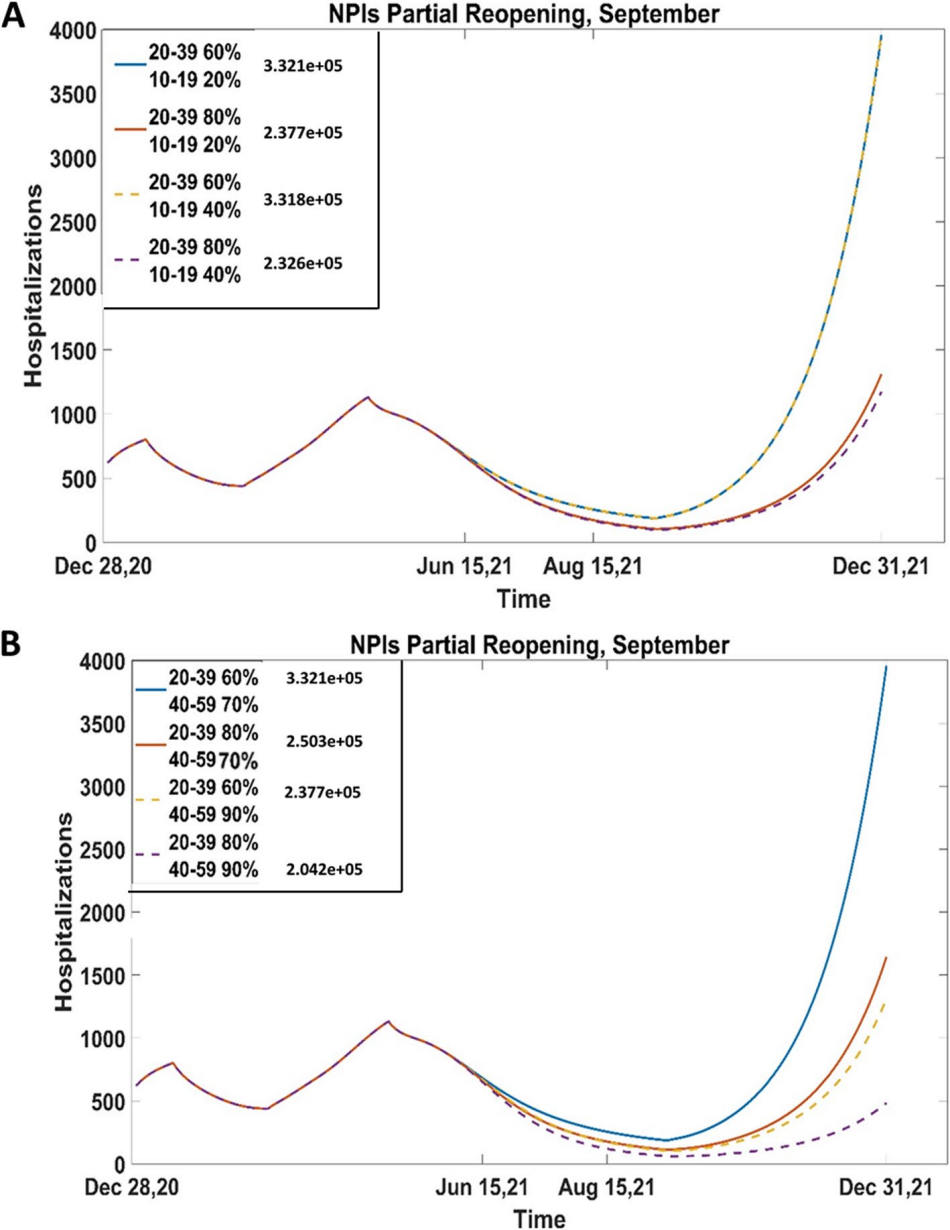
Hospitalizations with partial reopening in September (**A**) if 10–19 is vaccinated 20%-40%, 20–39 60%, 80% and 40–59, 60–79 and 80 + reached coverages 70%, 80%, 90%; (**B**) if 40–59 is vaccinated 70%-90%, 20–39 60%, 80% and 10–19, 60–79 and 80 + reached coverages 20%, 80%, 90%. The second dose is given at a rate of 1/112 days.^−1^. Cumulative cases are reported on the figure for reference. The projections of hospitalizations show that even a partial reopening in September will result in resurgence of the infection. From (**A**), we observe that vaccinating more teenagers and young adults is not statistically beneficial. On the other hand, even with lowest coverage of teenagers (**B**) if more adults are vaccinated, the hospitalizations from roughly 4000 to 500

Figure 3 shows hospitalizations under the scenario of partial reopening in September, if 60%-80% of the 20–39 age group is vaccinated and the 10–19 age group coverage is 20%-40% (A) or the 40–59 age group is 70%-90% (B). In both analyses, we observe an increasing trend of hospitalizations after the reopening, suggesting that a partial reopening strategy is not beneficial. Figure 3 also confirms what we observed in Fig. 2. It appears that the hospitalizations are not significantly reduced if teenagers are vaccinated; however, even with minimum coverage for age group 10–19 years, if age groups 20–39 years and 40–59 years are vaccinated to their maximum coverage, the hospitalization at the end of December will be about 500.

#### Identification of the best combination of vaccination coverages and NPIs lift dates and levels

**Fig. 4:**
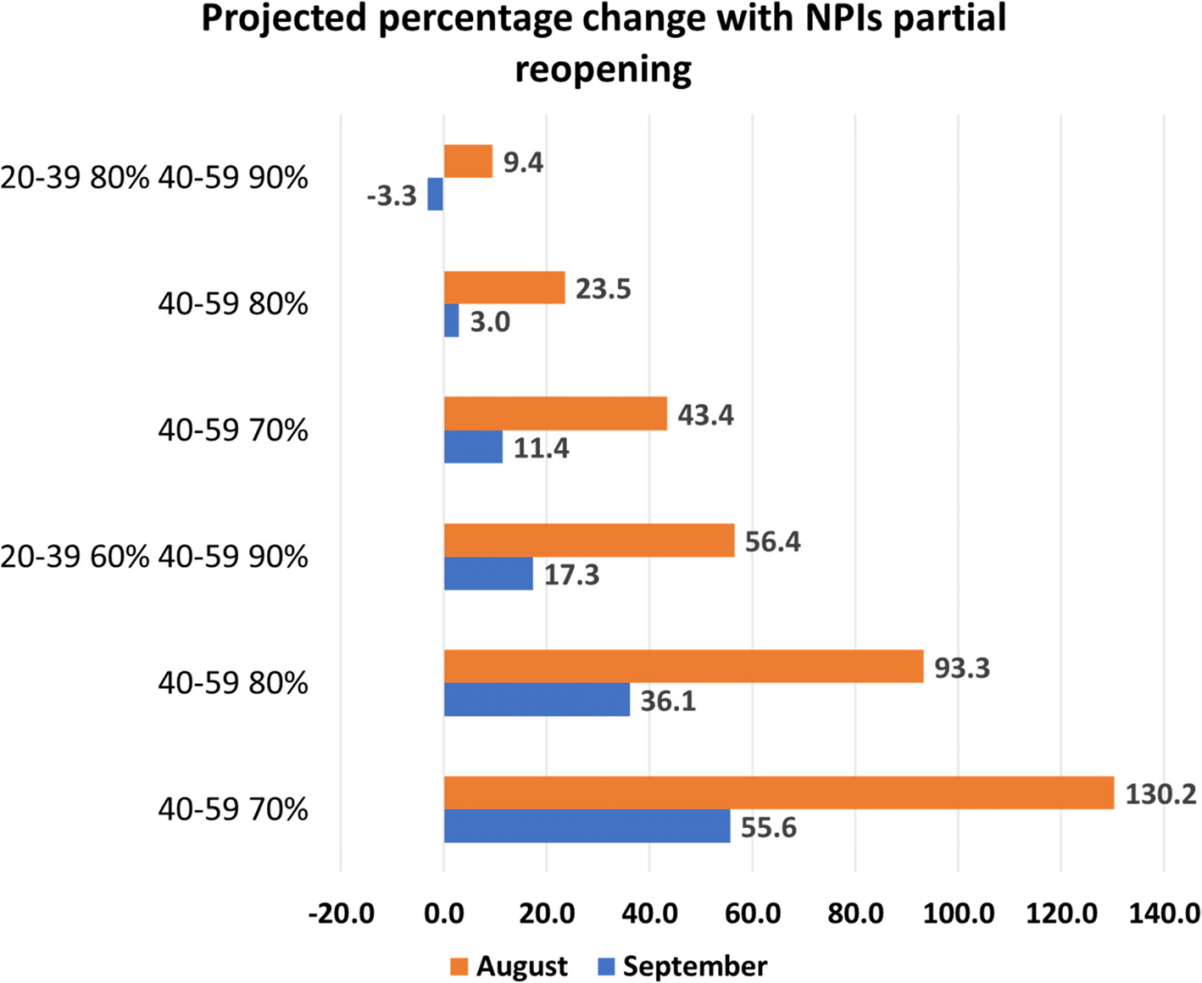
Percentage change of cumulative cases with respect to the baseline no reopening in SI Figure SI3 with partial reopening in August and September, when age groups 10–19, 60–79 and 80 + reach coverages 20%, 80%, 90%. The second dose is given at a rate of 1/112 days.^−1^. It is evident that reopening earlier will give a larger increase of cases, even with the highest coverage among adults

From Fig. 4, we immediately observe that if partial reopening occurs in August, cases increase up to 130.2% from the baseline, with a 9.4% increase in the scenario of highest vaccination coverage in the age groups 20–39 and 40–59 years. A partial late reopening is more beneficial than an early one, even with the lowest vaccine coverages (55.6% increase versus 130.2%). A similar pattern is shown with total reopening (Table SI5). On the other hand, we observe that with lifting NPIs to pre-pandemic levels (Table SI5), reopening in August is slightly more beneficial than reopening in September. This is due to the assumption of a fast-waning immunity rate for partially immunized individuals, whereas if reopening occurs later, more individuals become susceptible within the period of pre-reopening, and the infection spreads once NPI’s are lifted completely.

From Table SI5 and Table SI6, we also observe that a partial reopening gives the lowest increase of cases and deaths. As the transmission increases (due to higher number of contacts and/or higher probability of transmission like more transmissible variant), the percentage change escalates. This result is given if the reopening occurs in August or in September.

Projections of cumulative deaths show similar results of cumulative cases (Table SI6). However, with a pre-pandemic level reopening in September, if the coverage of 40–59 years age group is above 80%, the deaths are lower than the ones reported with reopening in August.

Hospitalizations are affected by the timing of lifting as well (Fig. 3B and Figure SI6). With minimal coverages in age groups 20–39 years and 40–59 years, the number of hospitalizations changes from 8000 to 4000 at the end of December, with partial reopening in August or in September, respectively. With maximum coverages of these age groups, reopening in September will drop the hospitalization on December 31, by roughly 50%.

#### Identification of the best combination of vaccination coverages and NPIs lift levels, with lower efficacy

**Fig. 5:**
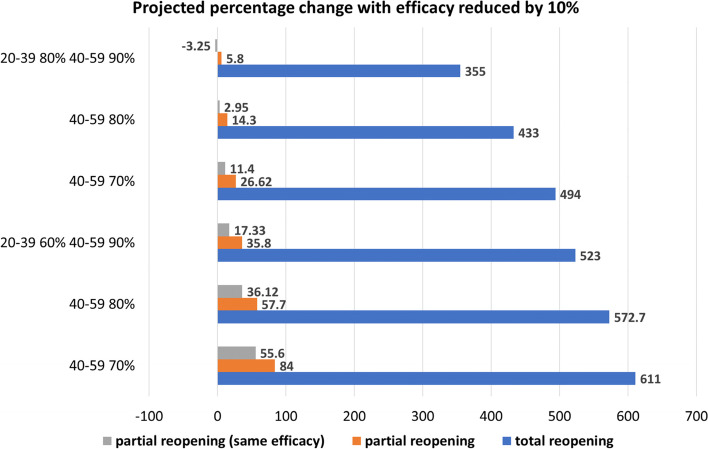
Percentage change of cumulative cases with respect to the base line no reopening in SI Figure SI3, reducing efficacy by 10%, with partial reopening when age groups 10–19, 60–79 and 80 + reach coverages 20%, 80%, 90%. A total reopening presents the highest increase compared to the partial reopening (both with lower efficacy and with the base line value). In general, if the vaccine is less efficient, the increase in cases is higher

With new variants circulating, the vaccine efficacy might be reduced. Figure 5 presents the percentage change of cumulative cases under different NPI lift levels, with the vaccine efficacy against the virus reduced by 10%. We observe that a lower efficacy leads to a large increase of cases, if compared to the highest efficacy analyzed. However, like the previous results, a partial reopening (orange bars) is much more beneficial than the total one (blue bars). Also, reopening to a pre-pandemic level (Table SI6) present the highest increase. However, it is important to mention that as the vaccination coverage of age group 20–39 and 40–59 years increase, cases decrease visibly (from a 84% to 5.8% increase for partial reopening, and 611% to 355% increase for total reopening).

A reduction of 10% in vaccine efficacy will result in an increase of hospitalizations from roughly 4000 to roughly 5500, with low coverage of vaccination of adults, and from roughly 500 to 1000, with highest coverage of these two groups (Figure SI7).

The percentage change of cumulative deaths is reported in Table SI7. Similar to the cases, a lower vaccine efficacy results in higher reported deaths.

#### Effect of reducing time between first and second dose

**Fig. 6:**
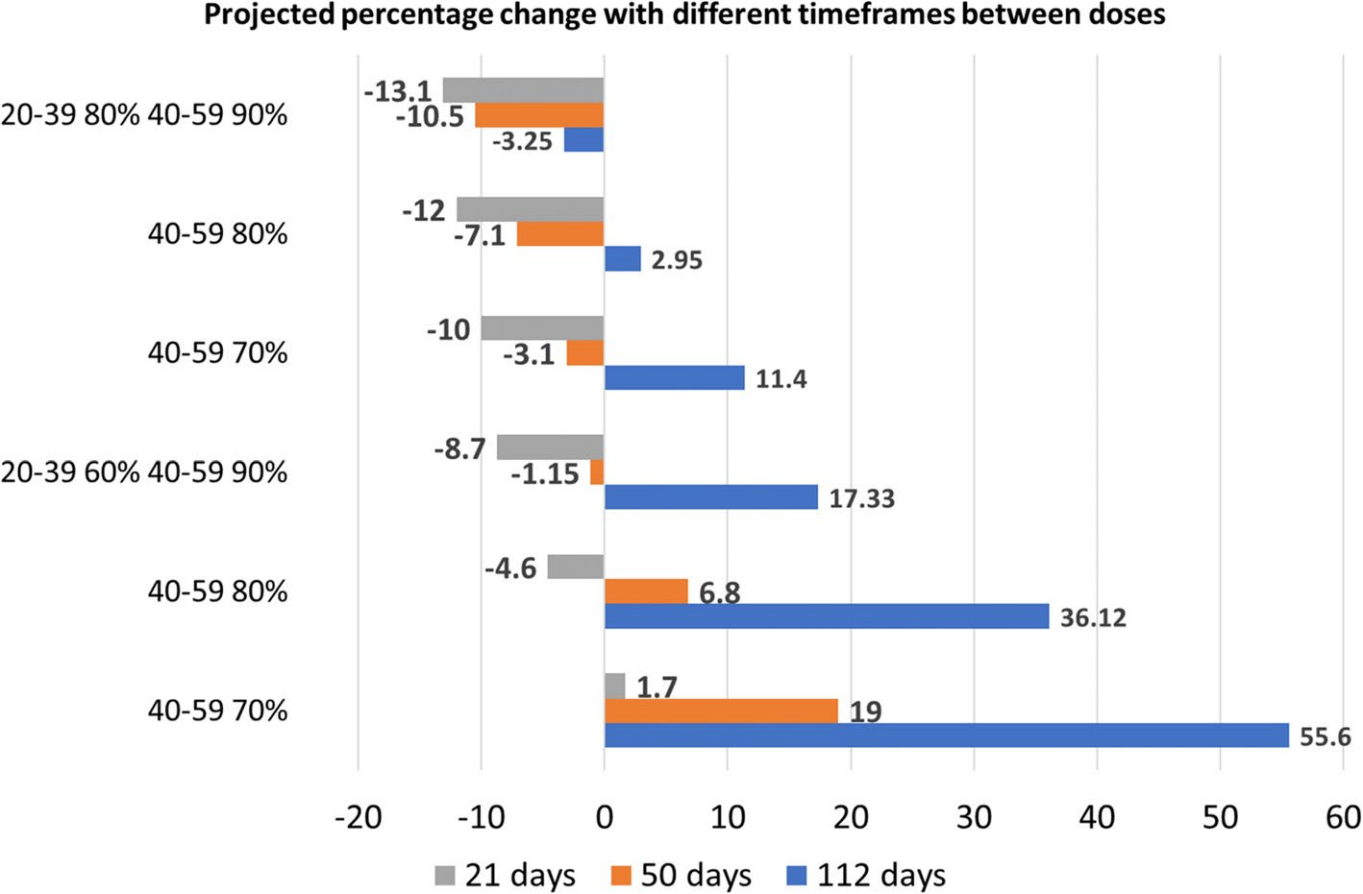
Percentage change of cumulative cases with respect to the base line no reopening in SI Figure SI3 with partial reopening in September and second dose given after 21, 50 or 112 days. Age groups 10–19, 60–79 and 80 + are assumed to reach coverages 20%, 80%, 90% by mid June 2021. We observe that reducing the time between the two doses is always beneficial. Also, with 21 days between doses, a decrease of cases is shown even if the age group 20–39 years reaches 60% of coverage, as long as the 40–59 years age group reaches 80%

Until the end of May 2021, in Ontario the second dose of vaccine was given after 16 weeks from the first one. Thereafter, this timeframe was shortened to 12 weeks [36]. According to the recommendation provided by the pharmaceutical manufacturing companies the two doses should be given after 21 days apart [31]. We compared how shortening the time needed to reach full immunization impacts the spread of the infection if the reopening level is partial (Fig. 6).

We observe that a faster rollout of second dose is always beneficial. With minimal coverage of age groups 40–59 years and 20–39 years, if the full immunization is reached after 21 days rather than 50 or 112, the percentage change of cases compared to the baseline drops from 55.6% to 19% to 1.7%. For the highest vaccination coverage, the increase is smaller than the one projected under the no reopening scenario, however a three-week gap between doses is still more beneficial. Also, a better control of the infection is possible even if the coverage of 20–39 years age group is 60% as long as the coverage of age group 40–59 years is 80%. Even with other reopening levels, the reduced time between doses appears to be more beneficial (Table SI8).

Minimizing the time between doses is also advantageous to reduce the number of deaths and hospitalizations (see Table SI9 and Figure SI8). With a partial reopening and minimum vaccine coverages among adults between 20 and 59 years, hospitalizations are decreased by roughly 60% and 85% if the second dose is given after 50 or 21 days respectively instead of 112 days. If the vaccination coverage is the highest, hospitalization at the end of December 2021 will be roughly 0 or 100, if the second dose is given after 21 or 50 days respectively.

### Sensitivity analysis

Sensitivity analysis conducted on the daily doses and rate at which the second dose is given shows that the model parameters having the highest impact on the cumulative cases, deaths, and hospitalizations 50 days after reopening are the vaccination rates of age groups 20–39 and 40–59 years and the time between doses (Table SI11). In particular, the PRCC values show negative correlation between these parameters and the model outcomes. This result suggests that not only adults need to be targeted to reduce cases, deaths, and hospitalizations, but also reducing the time between doses is beneficial. We also conducted sensitivity analysis of the infection-related parameters, such as number of contacts and age-dependent susceptibility on cumulative cases and deaths (Table SI12). The PRCC values show that contacts and susceptibility in ages 20–39 and 40–59 years have a significantly positive effect on the model outcomes.

## Discussion

We developed an age-structured compartmental model which captures the transmission dynamics of COVID-19. The SLAIHDR model considers vaccination and waning processes and an infectious compartment that captures both symptomatic and asymptomatic cases. Hospitalizations and deceased individuals are also included. The population is divided into six age groups and assumes that children aged 0 to 9 years are not immunized against the SARS-CoV-2 virus. Given the emergence of new variants, the growth of cases deriving from variants of concern (VOC) was captured using a time-dependent sigmoidal function. This needed to be included in the model to better predict the course of the infection and effectiveness of vaccines. This approach can identify severity differences between strains for outcomes such as death and hospitalization rates.

Our analysis shows that while prioritizing ages 10–19 years for vaccination rollout will not have a large impact, reaching 80% vaccine coverage in ages 20–39 and 40–59 by mid June 2021 will maximize reductions of cases, deaths, and hospitalizations. Sensitivity analysis confirms this result. Our results also confirm, as expected, that a late partial and total reopening will reduce the infection outcomes by roughly 57%; we still observe that the more adults aged between 20 and 59 years are vaccinated, the lower increase of cases and deaths is reported. However, even if delayed, a complete reopening, with the number of pre-pandemic contacts, will result in a visible spread of infection, also with the highest vaccine coverage.

As of June 14, 2021, the coverage in Toronto of adults is 76.12% and 72.9% for the age groups 20–39 and 40–59 years, respectively [39]. Over summer 2021 a strong immunization campaign was conducted, allowing more citizens to become fully immunized over a short period of time. However, after the reopening stages in August, a new wave, although smaller than the previous ones, was reported in the City of Toronto [26], followed by a much larger wave reported in late 2021 and early 2022 (Figure SI9). Our results suggest that with more relaxed NPIs in August or September, cases will increase towards the end of 2021, even with the highest vaccine coverages, and this confirms the visible increase in severe outcomes reported in Toronto after the reopening.

With new variants circulating, vaccine efficacy plays an important role in rollout strategies. Our analysis on the vaccine distribution and reopening strategies shows that with a lower efficacy against the virus, regardless of the reopening levels, the number of cases, deaths and hospitalizations reported increase. These results are confirmed in the new wave in December 2021 that resulted from the low efficacy of vaccine against the new variant Omicron and its higher transmissibility. Our result suggests that a prompt response of public health in increasing the immunization level is crucial if new variants circulate in the population. Moreover, our model suggests that even with a lower efficacy, it is important to vaccinate elderly and adults to minimize severe outcomes and infections.

The time at which NPIs are lifted has a substantial impact on the control of the infection. Our results show that in general, a late reopening, is more beneficial. In fact, with partial reopening in September rather than August, cases, deaths and hospitalizations are reduced.

Since the second vaccine dose increases efficacy, faster distribution of vaccine to reach full immunization can control the spread more quickly. Our analyses examining the impact of administering second doses after 21 or 50 days, show a higher reductions in case counts if full immunity is provided 3 weeks following the first dose. This result is expected from the formulation of our model, since a shorter period between doses will increase the number of individuals who are fully immunized faster.

Our study has some limitations. Firstly, we assumed that all the VOC cases are coming from B1.1.7 and the efficacy against the virus is the same for wildtype variants and VOC. However, as new variants emerge, with a much lower vaccine efficacy, it will be important in future work to consider multiple strains to better capture the role of efficacy and vaccine rollout. Secondly, we assumed that recovered individuals from any variant are not susceptible to other variants, but with more transmissible variants emerging, infection-acquired immunity might protect individuals only partially. Thirdly, while we assumed that all individuals vaccinated with the first dose will eventually receive the second dose, a fraction of people might opt not to receive the second dose. Lastly, we assumed that vaccination is effective from the day it is received, however individuals are considered fully immunized after 14 days from their last inoculation [40]. This study was motivated by the emergence and persistence of the first VOC, alpha. However, since our initial study the delta variant and later omicron variant emerged. Furthermore, additional doses of vaccine began being offered beyond the two-dose regiment considered in our model. The evolving nature of the virus and our societal response make it difficult to produce rapid real-time modelling that is both reflective of current realities and has accurate predictive power. Instead, this study considers how to effectively distribute vaccines between age groups when two strains of a virus are circulating. Though focused on alpha, the increased transmissibility of other variants and their increasing evasiveness of vaccine impact all ages and therefore the relative differences in disease impact between age groups is generally agnostic to variant. Nevertheless, we have compared our cases predictions with real cases data up to December 2021 in Figure SI9 which shows comparable qualitative trends between model and data. In fact, we observe that some of our predictions on cases, with low efficacy, high vaccine coverage and different reopening degrees, and hence higher transmission, reflect the level of cases reported at the end of 2021. We further note that the actual case data falls within our precited case counts for different reopening strategies. While the late surge in 2021 was due to omicron, a variant not considered in this model, the bounding from our model suggests that a re-parameterization of the model as new variants emerge can extend its validity and applicability.

## Conclusions

In conclusion, our model reflects the course of COVID-19 infection in Toronto considering infection from the VOC and original wildtype strain. We were able to capture, through data, the different infection outcomes such as transmission, hospitalizations, and deaths, generated by different variants of the virus. Our results show that it is imperative to direct our efforts towards individuals aged between 20 and 59 years, showing similarities with previous works [6, 22, 23] In fact, these are the age groups with higher contacts, social activity, and population size. Moreover, we showed that a complete return to the number of pre-pandemic contacts will result in an immediate virus resurgence, even with the highest vaccine coverages reached by mid June 2021. The situation may be even worse if there are new more transmissible variants. Also, we showed that the introduction of new variants, the vaccine efficacy against them, and the reduced time to obtain full immunity play an important role in the vaccination rollout aimed to reduced new infections and severe outcomes. A reduction in vaccine efficacy will lead to a higher spread of the infection, and if, additionally, the second dose is delayed too much, the risk of having a re-emergence of the infection is possible.

## Supplementary Information

**Additional file 1. **

## Data Availability

The datasets generated and/or analysed during the current study are available in the Covid_vaccine_NPIs_paper repository, https://github.com/EAruffo/Covid_vaccine_NPIs_paper.git Parameters used to generate analyses are provided in Supplementary Information.
