# Supplementary material for: Mathematical modelling of vaccination rollout and NPIs lifting on COVID-19 transmission with VOC: a case study in Toronto, Canada
Source: BMC Public Health. 2022 Jul 15;22:1349. doi: 10.1186/s12889-022-13597-9 (PMC9283818; doi:10.1186/s12889-022-13597-9)
Supplement: Supplementary file 1 — Additional file 1. [file 12889_2022_13597_MOESM1_ESM.pdf]

Supplementary Information

Mathematical modelling of vaccination rollout and NPIs lifting on COVID-19 transmission with VOC: a case study in Toronto, Canada

Elena Aruffo[1,2*], Pei Yuan[1,2*], Yi Tan[1,2*], Evgenia Gatov[3], Iain Moyles[1,2], Jacques Bélair[1,5], James Watmough[1,6], Sarah Collier[3], Julien Arino[1,4], Huaiping Zhu[1,2 †]

[1] Centre for Disease Modelling (CDM), York University, Toronto, Canada

[2] Department of Mathematics and Statistics, York University, Toronto, Canada

[3] Toronto Public Health, City of Toronto, Toronto, ON, Canada.

[4] Department of Mathematics, University of Manitoba, Winnipeg, Manitoba, Canada

[5] Département de Mathématiques et de Statistique, Université de Montréal, Montréal, Québec, Canada

[6] Department of Mathematics and Statistics, University of New Brunswick, Fredericton, New Brunswick, Canada

* Co-first authors made equal contributions.

† Corresponding Author: huaiping@yorku.ca,

4700 Keele Street, Toronto, Ontario, Canada, M3J1P3

Model

The system of ODEs describing the dynamic is given by:

$$
\begin{aligned}
S_i' &= -\lambda_{s_i} - \phi_i S_i \left\{ \left(1 - b(t)\right) \left[\beta^O \sum_j^6 c_{ij}(A_i + \xi I_{m_i}) \right] \right. \\
&\qquad \left. + \; b(t) \left[\beta^N \sum_j^6 c_{ij}(A_i + \xi I_{m_i}) \right] \right\} + \omega_1 V_{1i} + \omega_2 \mathrm{V}_{2\mathrm{i}} \\
L_i &= \phi_i S_i \left\{ \left(1 - b(t)\right) \left[\beta^O \sum_j^6 c_{ij}(A_i + \xi I_{m_i}) \right] - \; b(t) \left[\beta^N \sum_j^6 c_{ij}(A_i + \xi I_{m_i}) \right] \right\} \\
&\qquad + \phi_i V_{1i}\left(1 - \epsilon_{1_i}\right) \left\{ \left(1 - b(t)\right) \left[\beta^O \sum_j^6 c_{ij}(A_i + \xi I_{m_i}) \right] \right. \\
&\qquad \left. + \; b(t) \left[\beta^N \sum_j^6 c_{ij}(A_i + \xi I_{m_i}) \right] \right\} - \left(1 - b(t)\right)\alpha^o L_i - b(t)\alpha^N L_i \\
A_i' &= (1-\rho)\left(1 - b(t)\right)\alpha^o L_i + (1-\rho) b(t)\alpha^N L_i - \left(1 - b(t)\right)\gamma_{aR_i}^O \mathrm{A_i} \\
&\qquad - b(t)\gamma_{aR_i}^N \mathrm{A_i} \\
I'_{m_i} &= \rho\left(1 - b(t)\right)\alpha^o L_i + \rho b(t)\alpha^N L_i - \left(1 - b(t)\right)\gamma_{H_i}^o \mathrm{I_{m_i}} - b(t)\gamma_{H_i}^N \mathrm{I_{m_i}} \\
&\qquad - \left(1 - b(t)\right)\gamma_{mR_i}^O \mathrm{I_{m_i}} - b(t)\gamma_{mR_i}^N \mathrm{I_{m_i}} \\
H_i' &= \left(1 - b(t)\right)\gamma_{H_i}^o \mathrm{I_{m_i}} + b(t)\gamma_{H_i}^N \mathrm{I_{m_i}} - \left(1 - b(t)\right)\mu_{H_i}^o H_i - \; b(t)\mu_{H_i}^N H_i \\
&\qquad - \left(1 - b(t)\right)\gamma_{HR_i}^O H_i - b(t)\gamma_{HR_i}^N H_i \\
D_i'^o &= \left(1 - b(t)\right)\mu_{H_i}^o H_i + \; b(t)\mu_{H_i}^N H_i \\
R_i' &= (1 - b(t))\gamma_{aR_i}^O \mathrm{A_i} + b(t)\gamma_{aR_i}^N \mathrm{A_i} + \left(1 - b(t)\right)\gamma_{mR_i}^O \mathrm{I_{m_i}} + b(t)\gamma_{mR_i}^N \mathrm{I_{m_i}} \\
&\qquad + \left(1 - b(t)\right)\gamma_{HR_i}^O H_i + b(t)\gamma_{HR_i}^N H_i \\
V_{1i} &= \lambda_{s_i} - \phi_i V_{1i}\left(1 - \epsilon_{1_i}\right) \left\{ \left(1 - b(t)\right) \left[\beta^O \sum_j^6 c_{ij}(A_i + \xi I_{m_i}) \right] \right. \\
&\qquad \left. + \; b(t) \left[\beta^N \sum_j^6 c_{ij}(A_i + \xi I_{m_i}) \right] \right\} - \sigma\epsilon_{2_i} V_{1i} - \omega_1 \mathrm{V_{1i}} \\
V_{2i} &= \sigma\epsilon_{2_i} V_{1i} - \omega_2 \mathrm{V_{2i}}
\end{aligned}
$$

Eq.SI1

For $i \in \{1,2,3,4,5,6\}$, where $\beta^N = \zeta\beta^O$.

The list of variables and assumptions is given in Table SI1.

Table SI1: Table of the model's variables and assumptions

**Variable**	**Definition**
S_i	Susceptible individuals in age group i
L_i	Latently infected individuals in age group i
A_i	Asymptomatic individuals in age group i
I_{m_i}	Symptomatic (mild) individuals in age group i
H_i	Hospitalized individuals in age group i
D_i	Deceased individuals in age group i
R_i	Recovered individuals in age group i
V_{1_i}	Vaccinated individuals in age group i (first dose)
V_{2_i}	Vaccinated individuals in age group i (second dose)
$i \in \{1,2,3,4,5,6\}$	Age groups: 0-9,10- 19, 20-39, 40-59, 60-79, 80+ years respectively

**Assumptions**
1. Only susceptible individuals, aged 10 years and older, will receive the vaccine
2. Immunity follows two steps: partial (receiving one dose) and full (receiving two doses)
3. The vaccine efficacy is age-dependent (higher for teenagers and adults, lower for elderly)
4. The vaccine efficacy is the same against wildtype variant and VOC
5. The second dose is given after 112 days (in some predictive scenarios after 50 or 21 days), following the suggestion announced by the Government of Ontario in March 2021
6. Immunity wanes from one dose of vaccine after 120 days and from two doses after 365 days
7. We assume that the coverages in Table 2 are reached by June 14, 2021, and continue the vaccination process until 80% of the total population is vaccinated
8. We assume that all non-wild type cases belong to B.1.1.7
9. VOC and wildtype are both included in the transmission process, assuming that proportion of cases from VOC increases by time, following a sigmoidal function
10. The transmission from VOC is assumed to be 1.5 higher than the original variant
11. Vaccine reduces susceptibility. Partially vaccinated people can become infected and infectious if the vaccine is not efficient
12. Only individuals hospitalized might die from the infection

Table SI2: Table of model parameters

Parameter	Definition	Value		Ref.
λ_{s_i}	Average daily vaccine doses given at age group i	daily doses from data		**[1]**
ϕ_i	Susceptibility for age group i	0.34 | 0.34 | 1 | 1 | 1.67 | 1.67		**[2]**
c_{ij}	Contacts per day			**[3]**
	Reduction	0.671233066942591	Phase I	**Estimated**
		0.751639442206889	Phase II	
		0.693229501323643	Phase III	
		0.707785478752892	Phase IV	
β	Probability of transmission	1.87421367499059e-07		**Estimated**
ζ	Increase in transmission from VOC	1.5		**Assumed [4,5,6,7]**
ξ	Proportion of mild cases not adhering to self-isolation rule	0.225593198112631		**Estimated**
$\alpha^{O,N}$	average time in latent period	1/4 days^{-1}		**[8,9] (assumed for VOC)**
ρ	Proportion of symptomatic individuals	0.8		**[10]**
$\gamma_{aR_i}^{O,N}$	Recovery rate from asymptomatic infection	1/6 days^{-1}		**[11]**
$\gamma_{H_i}^{o}$	Hospitalization rate of individuals in group I, infected with old variant	0.0022 0.0004 0.0021 0.0082 0.0346 0.0760	Phase I	**Estimated**
		0.0015 0.0013 0.0030 0.0072 0.0302 0.0759	Phase II	
		0.0012 0.0010 0.0035 0.0106 0.0377 0.0926	Phase III	
		0.0010 0.0004 0.0024 0.0073 0.0235 0.0554	Phase IV	
$\gamma_{H_i}^{N}$		0 0 0 0 0.1927 0.0856	Phase I	

	Hospitalization rate of individuals in group I, infected with VOC	0.0017 0.0012 0.0042 0.0114 0.0579 0.1139	Phase II	**Estimated**
		0.0007 0.0010 0.0048 0.0167 0.0479 0.1232	Phase III	
		0.0014 0.0006 0.0041 0.0087 0.0306 0.0906	Phase IV	
$\gamma^{o}_{mR_i}$	Recovery rate of individuals in group I, mildly infected with old variant	0.0991 0.0998 0.0992 0.0968 0.0865 0.0704	Phase I	**Calculated**
		0.0993 0.0994 0.0986 0.0967 0.0860 0.0649	Phase II	
		0.0995 0.0996 0.0985 0.0955 0.0841 0.0608	Phase III	
		0.0996 0.0998 0.0990 0.0970 0.0902 0.0768	Phase IV	
$\gamma^{N}_{mR_i}$	Recovery rate of individuals in group I, mildly infected with VOC	0.1000 0.1000 0.1000 0.1000 0.0250 0.0667	Phase I	**Calculated**
		0.0992 0.0994 0.0981 0.0947 0.0732 0.0473	Phase II	
		0.0997 0.0996 0.0980 0.0929 0.0798 0.0479	Phase III	
		0.0994 0.0997 0.0983 0.0964 0.0872 0.0621	Phase IV	
$\mu^{o}_{H_i}$	Mortality rate from old variant	0 0 0.0028 0.0067 0.0174 0.0463	Phase I	**Estimated**
		0 0 0.0012 0.0068 0.0169 0.0284	Phase II	
		0 0 0.0080 0.0049 0.0129 0.0279	Phase III	
		0 0 0 0.0022 0.0016 0.0095	Phase IV	
$\mu^{N}_{H_i}$	Mortality rate from VOC	0 0 0 0 0.0241 0	Phase I	**Estimated**
		0 0 0.0045 0.0063 0.0170 0.0484	Phase II	
		0 0 0.0020 0.0037 0.0122 0.0309	Phase III	
		0 0 0 0 0.0025 0.0074	Phase IV	
$\gamma^{O}_{HR_i}$	Recovery rate of hospitalized individuals in group I, mildly infected with old variant	0.1073 0.1073 0.1031 0.0974 0.0815 0.0386	Phase I	**Estimated**
		0.1535 0.1535 0.1506 0.1379 0.1146 0.0883	Phase II	
		0.1233 0.1233 0.1136 0.1174 0.1077 0.0895	Phase III	
		0.1034 0.1034 0.1034 0.1012 0.1018 0.0937	Phase IV	
$\gamma^{N}_{HR_i}$	Recovery rate of hospitalized individuals in group I, mildly infected with VOC	0.1073 0.1073 0.1073 0.1073 0.0715 0.1073	Phase I	**Estimated**
		0.1535 0.1535 0.1432 0.1391 0.1146 0.0423	Phase II	
		0.1233 0.1233 0.1209 0.1188 0.1085 0.0859	Phase III	
		0.1034 0. 0.1034 0.1034 0.1009 0.0959	Phase IV	
ϵ_{1_i}	Efficacy first dose for age group i	0 0.8 0.8 0.8 0.7 0.7<br>reduced by 0.1 in lower efficacy scenario		**Assumed [12]**
ϵ_{2_i}	Efficacy second dose for age group i	0 0.9 0.9 0.9 0.8 0.8<br>reduced by 0.1 in lower efficacy scenario		**Assumed [12]**

σ	Average time to receive second dose	1/112 $days^{-1}$	[13]
ω_1	Average time to wane immunity after first dose	1/120 $days^{-1}$	**Assumed**
ω_2	Average time to wane immunity after second dose	1/365 $days^{-1}$	**Assumed**
S_{0_i}	Susceptible individuals in age group I (initial value)	283101 279614 895972 826657 544565 150006	**Calculated**
E_{0_i}	Exposed individuals in age group I (initial value)	158 301 1529 1171 555 265	**Calculated**
A_{0_i}	Asymptomatic individuals in age group I (initial value)	46 78 340 300 152 62	**Calculated**
I_{m0_i}	Symptomatic individuals in age group I (initial value)	340 529 2378 1936 877 318	**Calculated**
H_{0_i}	Hospitalized individuals in age group I (initial value)	4 4 44 121 236 210	**Calculated**
D_{0_i}	Deceased individuals in age group I (initial value)	1 0 4 79 527 1351	**Calculated**
R_{0_i}	Recovered individuals in age group I (initial value)	2735 4637 21635 17060 8010 3588	**Calculated**
$V_{1_{0i}}$	Partially vaccinated individuals in age group I (initial value)	0 15 1309 2038 597 271	**Calculated**
$V_{1_{0i}}$	Vaccinated individuals in age group I (initial value)	0 0 0 0 0 0	**Calculated**

SENSITIVITY ANALYSIS PARAMETERS

**PARAMETER**	**DEFINITION**	**RANGE (uniform distribution)**

σ	Rate at which second dose is distributed	[1/112, 1/21]
λ_2	Daily doses age group 10-19	[500, 2719]
λ_3	Daily doses age group 20-39	[1624, 8559]
λ_4	Daily doses age group 40-59	[2312, 8714]
λ_5	Daily doses age group 60-79	[599, 2702]
λ_6	Daily doses age group 80+	[319, 900]
c_{inc}	Percentage increase of contacts	[0.1, 1]
ϕ_i	Susceptibility of age group $i \in \{1,2,3,4,5,6\}$	[0.001, 1.8]

Proportion of VOC cases

To capture the increasing trend of cases from VOC, we defined a time-dependent function ($b(t)$) following a sigmoid function. Fig. A1 shows the proportion of cases from VOC from data (red circles) and the function used to reproduce their trend (blue curve). According to data up to May 19, 2021 the proportion of cases from VOC in Toronto reached a maximum of 0.8 by May 11, 2021. Hence, we consider 80% to be the maximum of cases generated by the new variant.

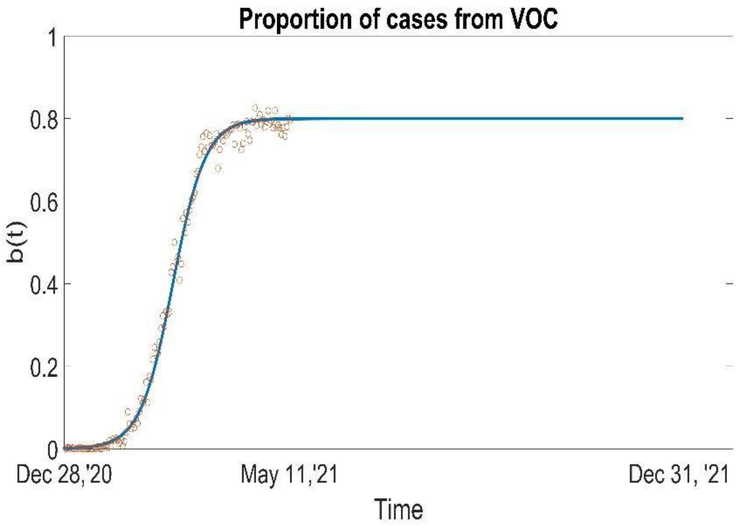

Figure SI1: Sigmoidal function describing the growth of proportion of cases from VOC in Toronto. Scatter plot represents the proportion of VOC cases in Toronto from December 28, 2020 to May 11, 2021.

Data fitting

To calibrate the model's parameters, we employed cumulative and daily cases and deaths, and hospitalizations (**Figure SI2**). Using the Latin Hypercube Sampling (LHS), we generated 500 samples for the initial guess of each parameter using a normal distribution. Then, for each initial guess of parameter set, employed the fmincon function in the MATLAB optimization Toolbox[14] to find the local minimum of the sum of squared differences between observed data and the model's estimates of daily confirmed cases and deaths, cumulative cases and deaths and hospitalizations. After finding the best parameter set for each sample, we evaluated the mean value and the standard deviation, obtaining the confidence interval where our parameters lie.

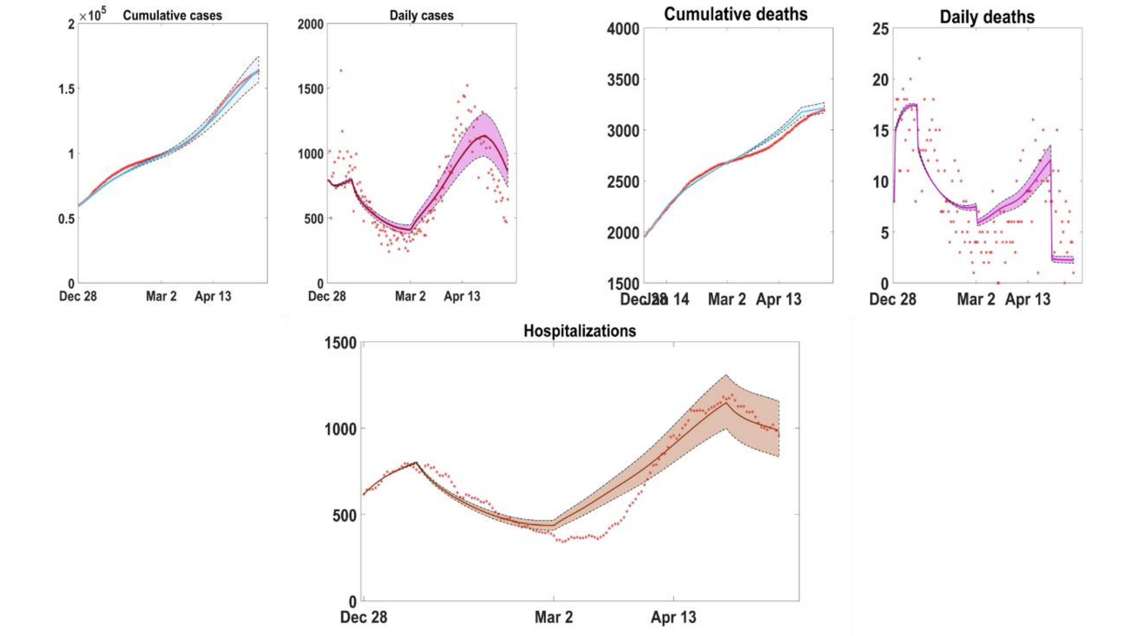

Figure SI2: Calibration of parameters calibration using Least Square Method. We used cumulative and daily cases and deaths, and hospitalizations between December 28, 2020 and May 19, 2021. Red line indicates the mean value; Blue and yellow lines indicate the upper and lower bound of the confidence interval.

Permutations of model's analysis

All the scenarios used for the projections are shown in Figure SI3. Each scenario is described by taking one element in each column.

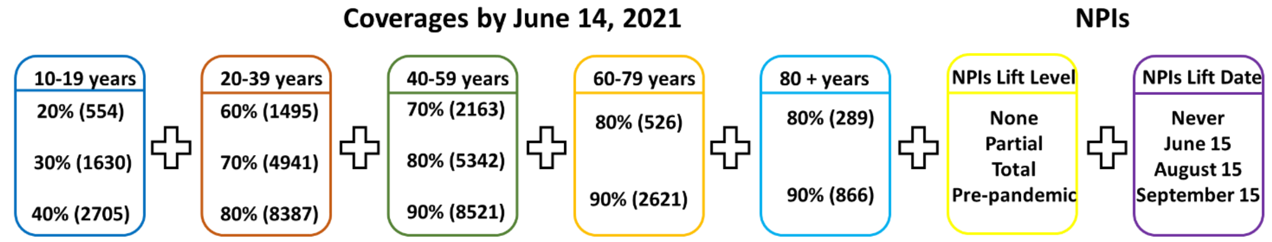

Baseline for analyses						
10-19 years	20-39 years	40-59 years	60-79 years	80 + years	NPIs Lift Level	NPIs Lift Date
20%	60%	70%	80%	80%	None	Never

Figure SI3: Outward-facing model coverages and base line for model's analysis. All these coverages are reached by June 14, 2021. In brackets, we report the daily doses. Each scenario is described by taking one element in each column.

Uncertainty of the parameters

Figure SI4: Variation of hospitalizations with respect to the parameters estimated in the confidence interval

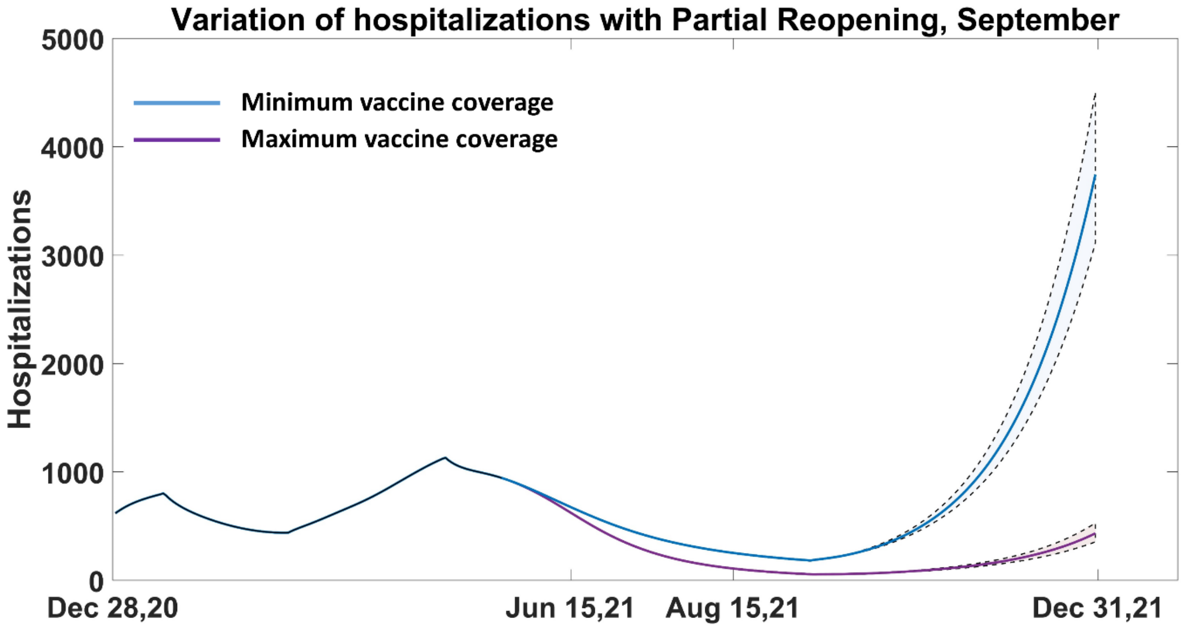

Contact matrix

We used the total contact matrix from a recent Canadian study[3]. However, the age groups used in this study were defined by a 5-year band from 0 to 80+. Our model is using larger age groups, then it was necessary to aggregate the original contact matrix in less groups.

Let's define P_j the population size of age group $j \in \{1,2,3, \dots 17\}$, where $1 = 0 - 4\, years, 2 = 5 - 9\, years, \dots, 17 = 80 +$ years. To better approximate the contact rates, we calculated, from the original 17x17 matrix (M_{ij}), the total contacts that an age group has with all the other age groups. To obtain this, we multiplied all the age groups by their own population size, i.e. $m_{ij} \times P_j$. Then, to aggregate some age groups, we averaged the total contacts as follows:

- For same ages belonging to new aggregation: we summed up the diagonal entries of the submatrix related to the age groups to aggregate and the average of the mixed contacts $\left(\hat{c}_{ii} = \sum m_{ii} + \sum \frac{m_{ij}+m_{ji}}{2}\right)$. For example, the new contact of the aggregated group 0-9, given by group 1 and 2, will be $m_{11} + m_{22} + \frac{m_{12}+m_{21}}{2}$

- For different ages aggregation: we summed up the average of the mixed contacts $\left(\hat{c}_{ij} = \sum \frac{m_{ij}+m_{ji}}{2}\right)$. For example, the new contact of the aggregated group 0-9 and 10-19, given by group 1, 2, 3 and 4, will be $\frac{m_{13}+m_{31}}{2} + \frac{m_{14}+m_{41}}{2} + \frac{m_{23}+m_{32}}{2} + \frac{m_{24}+m_{42}}{2}$

Once we reduced the total contacts into a smaller matrix, we re-parametrized each entry of the new age group dividing the obtained contacts by the population size of the aggregate age group (i.e., $c_{ij} = \hat{c}_{ij}/\sum P_j$). Table SI3 represents the compacted matrix.

Table SI3: Contact matrix

		**Age participants**					
		**0-9**	**10-19**	**20-39**	**40-59**	**60-79**	**80+**
**Age contacts**	**0-9**	2.61	0.55	0.59	0.73	0.22	0.04
	**10-19**	0.58	3.28	0.77	0.95	0.22	0.11
	**20-39**	2.23	2.72	3.35	3.82	1.50	0.66
	**40-59**	2.12	2.56	2.94	2.49	1.65	1.12
	**60-79**	0.41	0.39	0.75	1.08	1.22	1.05
	**80+**	0.02	0.05	0.09	0.20	0.29	0.58

RESULTS

Reproduction number R_c

Figure SI5: Contour plots of R_c assuming that the following coverages reached for age groups 10-19, 60-79 and 80+ years are 20%, 80%, and 90%, respectively, when the NPIs level reopening is (A) none, (B) partial, (D) total and (E) pre-pandemic. As expected, as the vaccination coverage increases, the values of the reproduction number decrease. Also, we observe that with the lowest reopening level, to reduce the reproduction number below 1, it is sufficient to vaccinate age groups 20-39 and 40-59 years above 60% and 62%, respectively. On the other hand, a relaxation of NPIs and increase in contacts as in NPIs partial reopening, the R_c will always be greater than 1. Similar results, but higher $\boldsymbol{R_c}$, are shown with NPIs pre-pandemic reopening (C).

A

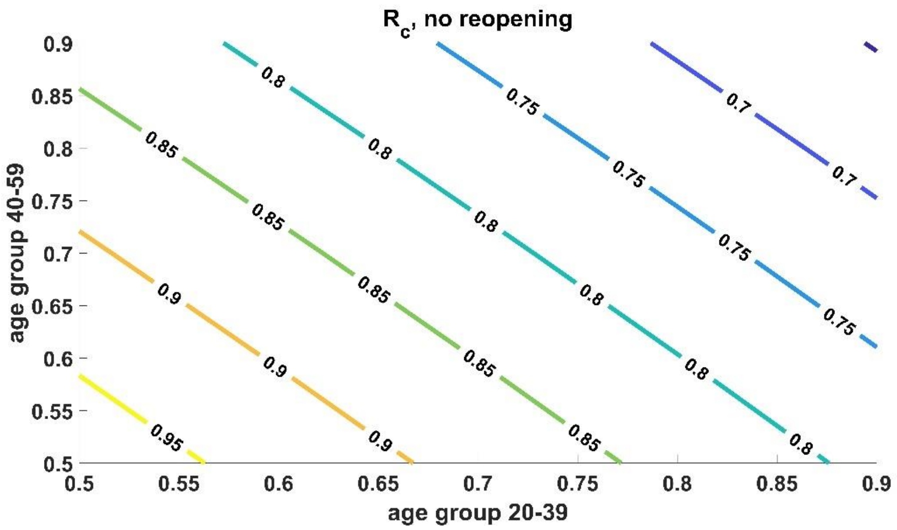

B

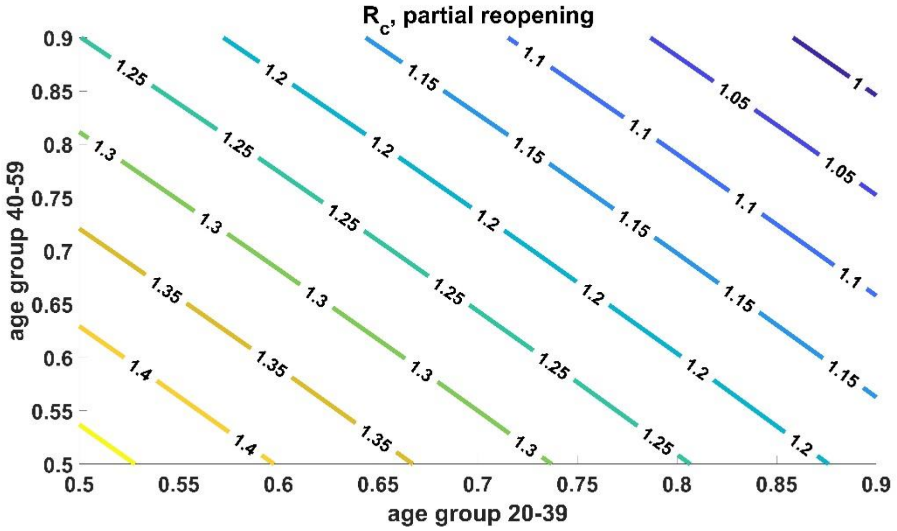

C

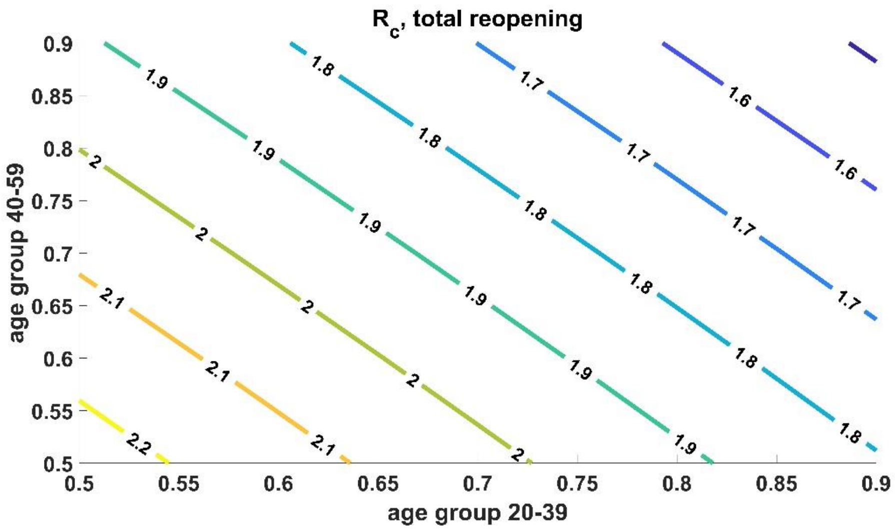

R_c, total reopening
age group 40-59
age group 20-39

D

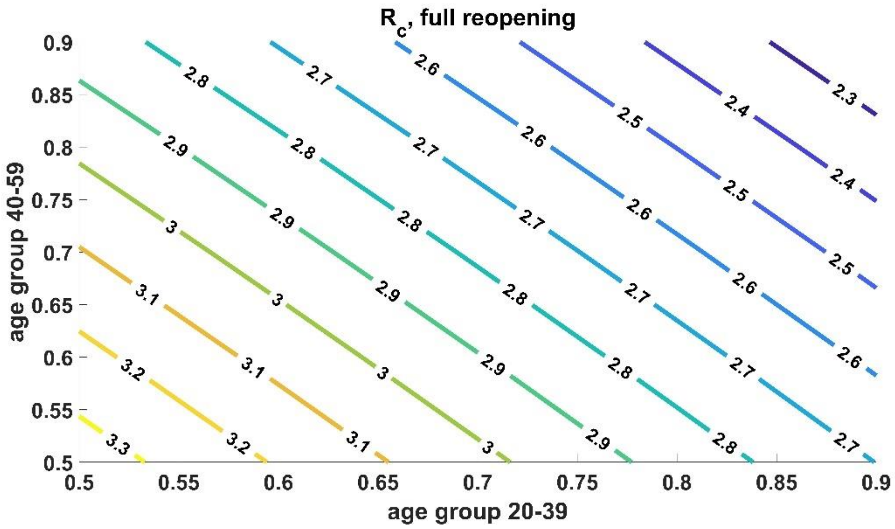

R_c, full reopening
age group 40-59
age group 20-39

Projections

Identification of age group that minimizes cases, deaths and hospitalizations

Table SI4: Percentage change of cumulative cases and deaths with respect to the base line NPIs no reopening in SI Figure SI3 with partial reopening in September, when age groups 60-79 and 80+ reached coverages 80%, 90% by June 14. Cases and deaths are reported comparing different coverages for age group 10-19 years, assuming 40-59 years fixed at 70% coverage (top table) and comparing different coverages for age group 40-59 years, assuming 10-19 years fixed at 20% coverage (bottom table). The second dose is given at a rate of 1/112 days^{-1}

**Projected percentage change of cumulative cases with respect to baseline NPIs no reopening in SI Figure SI3 after reopening in September with NPIs partial reopening**				**Projected percentage change of cumulative deaths with respect to baseline NPIs no reopening in SI Figure SI3 after reopening in September with NPIs partial reopening**			
		**20-39 years coverage by June 14, 2021**				**20-39 years coverage by June 14, 2021**	
		**60%**	**80%**			**60%**	**80%**
**10-19 years coverage by June 14, 2021**	**20%**	55.6	11.4	**10-19 years coverage by June 14, 2021**	**20%**	51.5	17.1
	**30%**	56.7	11.1		**30%**	52.4	17
	**40%**	55.5	9.03		**40%**	51.6	15.4
		**20-39 years coverage by June 14, 2021**				**20-39 years coverage by June 14, 2021**	
		**60%**	**80%**			**60%**	**80%**
**40-59 years coverage by June 14, 2021**	**70%**	55.6	11.4	**40-59 years coverage by June 14, 2021**	**70%**	51.5	17.14
	**80%**	36.12	2.95		**80%**	36.2	10.58
	**90%**	17.33	-3.25		**90%**	21.45	5.72

Identification of the best combination of vaccination coverages and NPIs lift dates

Table SI5: Percentage change of cumulative cases with respect to the baseline NPIs no reopening in SI Figure SI3 with partial, total and pre-pandemic reopening in August and September, when age groups 10-19, 60-79 and 80+ reached coverages 20%, 80%, 90%. The second dose is given at a rate of 1/112 days^{-1}.

**Projected percentage change of cumulative cases with respect to the base line NPIs no reopening in SI Figure SI3**							
		**In reopen in AUGUST**					
		**20-39 years coverage by June 14, 2021**					
		**60%**			**80%**		
**NPI's Level of reopening**		**Partial**	**Total**	**Pre-pandemic**	**Partial**	**Total**	**Pre-pandemic**
**40-59 years coverage by June 14, 2021**	**70%**	**130.2**	632	752	**43.43**	578	725
	**80%**	**93.25**	614	741	**23.5**	544	712
	**90%**	**56.4**	586	725	**9.42**	498.5	698
		**In reopen in SEPTEMBER**					
		**20-39 years coverage by June 14, 2021**					
		**60%**			**80%**		
**NPI's Level of reopening**		**Partial**	**Total**	**Pre-pandemic**	**Partial**	**Total**	**Total**
**40-59 years coverage by June 14, 2021**	**70%**	55.6	573	769.1	11.4	427	739.7
	**80%**	36.12	533	758	2.95	345	723.8
	**90%**	17.33	460	741.2	-3.25	256	705.5

Table SI6 : Percentage change of cumulative deaths with respect to the base line NPIs no reopening in SI Figure SI3 with partial, total and pre-pandemic reopening in August and September, when age groups 10-19, 60-79 and 80+ reached coverages 20%, 80%, 90%. The second dose is given at a rate of 1/112 days^{-1}.

**Projected percentage change of cumulative deaths with respect to the base line NPIs no reopening in SI Figure SI3**		
	**If reopen in AUGUST**	
	**20-39 years coverage by June 14, 2021**	
	**60%**	**80%**

NPI's Level of reopening		Partial	Total	Pre-pandemic	Partial	Total	Pre-pandemic
40-59 years coverage by June 14, 2021	70%	**127.3**	872	1115	**49.7**	774.7	1116
	80%	**93.1**	842.6	1113	**31.9**	698	1108
	90%	**60.1**	784.8	1103	**19.4**	604.8	1098
		**In reopen in SEPTEMBER**					
		**20-39 years coverage by June 14, 2021**					
		**60%**			**80%**		
**NPI's Level of reopening**		**Partial**	**Total**	**Pre-pandemic**	**Partial**	**Total**	**Pre-pandemic**
40-59 years coverage by June 14, 2021	70%	**51.5**	633.5	1132	**17.14**	396.7	1093
	80%	**36.2**	560.7	1119	**10.6**	295.4	1059
	90%	**21.4**	440.6	1091	**5.72**	204.8	1012

Figure SI6: Hospitalizations with partial reopening in August (A) if 40-59 is vaccinated 70%-00%, 20-39 60%, 80% and 10-19, 60-79 and 80+ reached coverages 20%, 80%, 90%. Cumulative cases are reported for reference. The second dose is given at a rate of 1/112 days^{-1}.

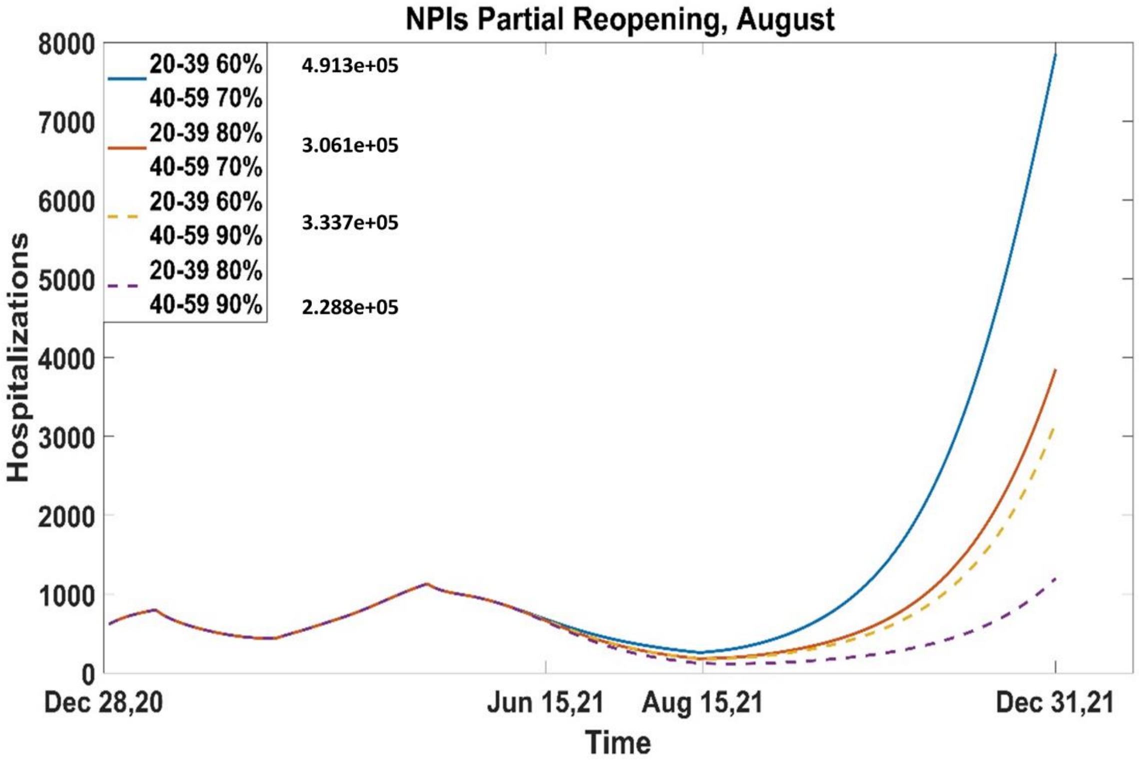

Identification of the best combination of vaccination coverages and NPIs lift date, with lowest efficacy

Table SI7: Percentage change of cumulative cases with respect to the base line NPIs no reopening in SI Figure SI3, reducing efficacy by 10%, with partial, total and pre-pandemic reopening when age groups 10-19, 60-79 and 80+ reached coverages 20%, 80%, 90%.

**Projected percentage change of cumulative cases with respect to the base line NPIs no reopening in SI Figure SI3 with reopening in September and efficacy reduced by 10%**							
		**20-39 years coverage by June 14, 2021**					
		**60%**			**80%**		
**NPI's Level of reopening**		**Partial**	**Total**	**Pre-pandemic**	**Partial**	**Total**	**Pre-pandemic**
**40-59 years coverage by June 14, 2021**	**70%**	**84**	611	784	**26.62**	494	756
	**80%**	**57.7**	572.7	772	**14.3**	433	742
	**90%**	**35.8**	523	759	**5.8**	355	727

Table SI8: Percentage change of cumulative deaths with respect to the base line NPIs no reopening in SI Figure SI3, reducing efficacy by 10%, with partial, total and pre-pandemic reopening when age groups 10-19, 60-79 and 80+ reached coverages 20%, 80%, 90%.

**Projected percentage change of cumulative deaths with respect to the base line NPIs no reopening in SI Figure SI3 with reopening in September and efficacy reduced by 10%**							
		**20-39 years coverage by June 14, 2021**					
		**60%**			**80%**		
**NPI's Level of reopening**		**Partial**	**Total**	**Pre-pandemic**	**Partial**	**Total**	**Pre-pandemic**
**40-59 years coverage by June 14, 2021**	**70%**	**76**	728.3	1173	**29.8**	509.2	1144
	**80%**	**54.5**	648.7	1161	**19.7**	417.2	1119
	**90%**	**36.5**	553.9	1142	**12.9**	315.2	1086

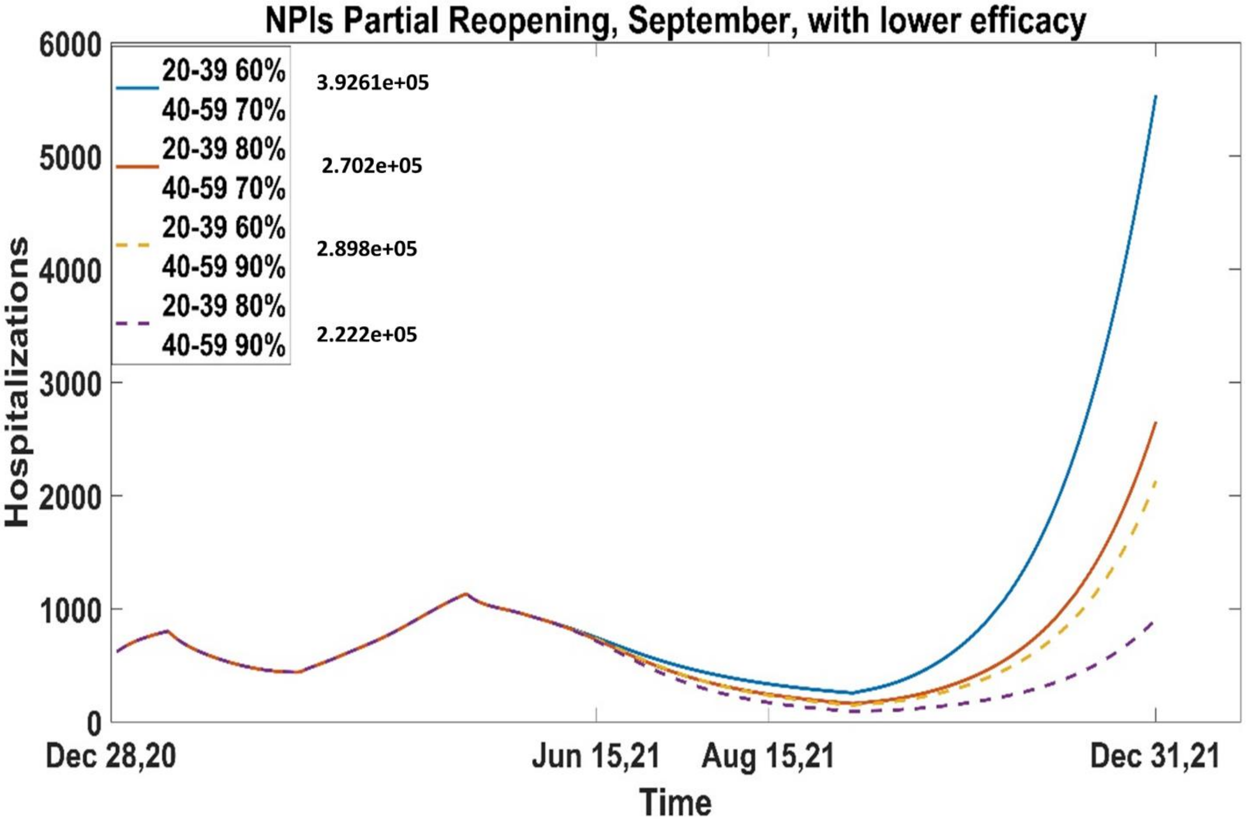

Figure SI7 : Hospitalizations if 40-59 is vaccinated 70%-90%, 20-39 60%, 80% and 10-19, 60-79 and 80+ reached coverages 20%, 80%, 90% with total NPIs reopening in September with efficacy decreased by 10%. Cumulative cases are reported for reference.

Effect of reducing time between first and second dose

Table SI9: Percentage change of cumulative cases with respect to the base line NPIs no reopening in SI Figure SI3 with partial, total and pre-pandemic reopening in September and second dose given after 21 or 50 days. Age groups 10-19, 60-79 and 80+ are assumed to reach coverages 20%, 80%, 90% by mid June. Par.= partial; Tot.= total; Pre-pan.= pre-pandemic.

**Projected percentage change of cumulative cases with respect to the base line NPIs no reopening in SI Figure SI3 with reopening in September**													
		**21 days Between dose 1 and dose 2**						**50 days Between dose 1 and dose 2**					
		**20-39 years coverage by June 14, 2021**						**20-39 years coverage by June 14, 2021**					
		**60%**			**80%**			**60%**			**80%**		
**NPI's Level of reopening**		**Par.**	**Tot.**	**Pre-pan.**	**Par.**	**Tot.**	**Pre-pan.**	**Par.**	**Tot.**	**Pre-pan.**	**Par.**	**Tot.**	**Pre-pan.**
**40-59 years coverage by June 14, 2021**	**70%**	**1.7**	284	659	**-10**	**58**	573	**19**	438	706	**-3.1**	215	662
	**80%**	**-4.6**	184	632.5	**-12**	**16.5**	494	**6.8**	347	686	**-7.1**	**135**	635
	**90%**	**-8.7**	**92.5**	592.7	**-13.1**	**-0.14**	396	**-1.15**	250	664	**-10.5**	54.4	582

Table SI10: Percentage change of cumulative deaths with respect to the base line NPIs no reopening in SI Figure SI3 with partial, total and pre-pandemic reopening in September and second dose given after 21 or 50 days. Age groups 10-19, 60-79 and 80+ are assumed to reach coverages 20%, 80%, 90% by mid June. Par.= partial; Tot.= total; Pre-pan.= pre-pandemic.

**Projected percentage change of cumulative deaths with respect to the base line NPIs no reopening in SI Figure SI3 with reopening in September**													
		**21 days Between dose 1 and dose 2**						**50 days Between dose 1 and dose 2**					
		**20-39 years coverage by June 14, 2021**						**20-39 years coverage by June 14, 2021**					
		**60%**			**80%**			**60%**			**80%**		
**NPI's Level of reopening**		**Par.**	**Tot.**	**Pre-pan.**	**Par.**	**Tot.**	**Pre-pan.**	**Par.**	**Tot.**	**Pre-pan.**	**Par.**	**Tot.**	**Pre-pan.**
**40-59 years coverage by June 14, 2021**	**70%**	**6.98**	207.8	818	**-0.61**	**43.2**	622.8	**20.5**	395	958.7	**4.82**	163	879.5
	**80%**	**2.78**	126.5	763.6	**-1.74**	**17.1**	463.1	**11.7**	284	926.4	**1.94**	**100**	810.5
	**90%**	**0.13**	**63.5**	668	**-2.31**	**6.67**	317.1	**6**	190	880.1	**-0.37**	**43.6**	673

Figure SI8: Hospitalizations with partial reopening in September if 40-59 is vaccinated 70%-00%, 20-39 60%, 80% and 10-19, 60-79 and 80+ reached coverages 20%, 80%, 90% and if the second dose is given at a rate of (A) 1/21 days^{-1} or (B) 1/50 days^{-1}. Cumulative cases are reported for reference.

A

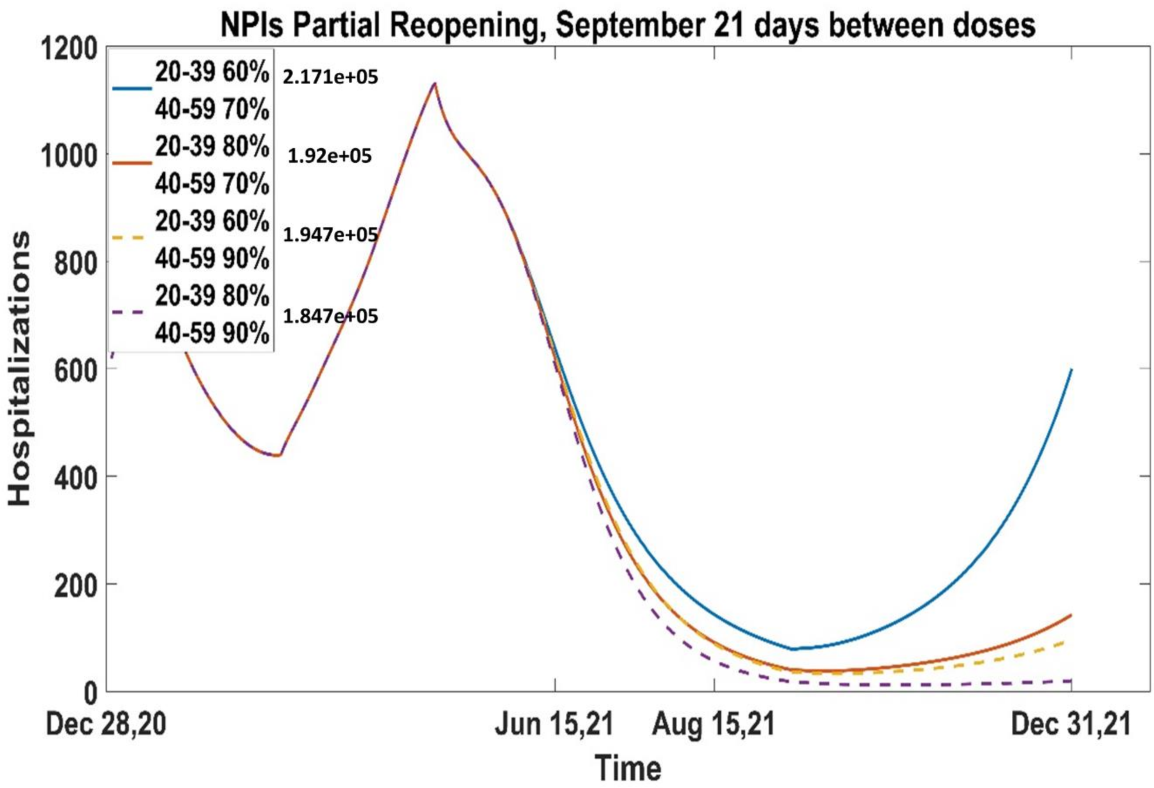

B

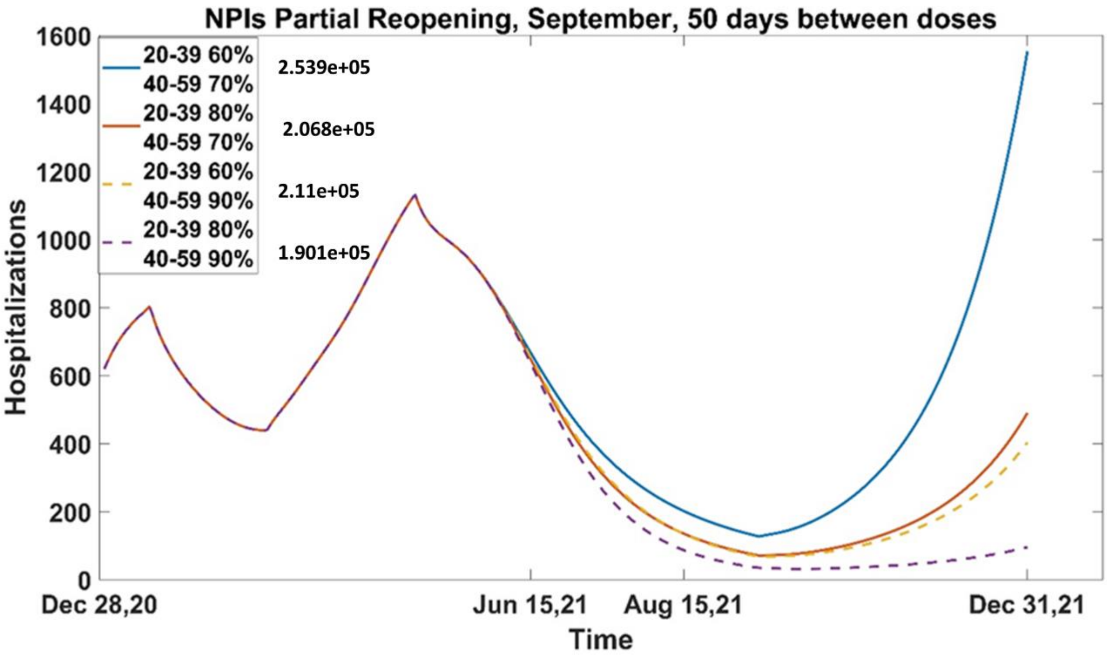

Sensitivity Analysis

Using the Latin Hypercube Sampling/Partial Rank Correlation Coefficient (*LHS*/*PRCC*) we conducted sensitivity analysis on the parameters related to vaccination as well as infection-related parameters.

Table SI11: PRCC on cumulative cases and deaths, investigating vaccine-related parameters.

**SENSITIVITY ANALYSIS**				
**PARAMETERS**	**DEFINITION**	**PRCC**		
		CASES	DEATHS	HOSPITALIZATION (50 days after reopening in June)
σ	Rate at which second dose is distributed	**-0.9409**	**-0.9409**	**-0.9638**
λ_2	Daily doses age group 10-19	0.01411	0.01411	0.02773
λ_3	Daily doses age group 20-39	**-0.8897**	**-0.8897**	**-0.923**
λ_4	Daily doses age group 40-59	**-0.8206**	**-0.8206**	**-0.9088**
λ_5	Daily doses age group 60-79	-0.1792	-0.1792	-0.4888
λ_6	Daily doses age group 80+	-0.03836	-0.03836	-0.1357
**SENSITIVITY ANALYSIS**				
**PARAMETERS**	**DEFINITION**	**PRCC**		
		CASES	DEATHS	HOSPITALIZATION (50 days after reopening in September)
σ	Rate at which second dose is distributed	**-0.9409**	**-0.9409**	**-0.9638**
λ_2	Daily doses age group 10-19	0.01411	0.01411	0.02773
λ_3	Daily doses age group 20-39	**-0.8897**	**-0.8897**	**-0.923**
λ_4	Daily doses age group 40-59	**-0.8206**	**-0.8206**	**-0.9088**
λ_5	Daily doses age group 60-79	-0.1792	-0.1792	-0.4888
λ_6	Daily doses age group 80+	-0.03836	-0.03836	-0.1357

Table SI11 shows the PRCCs of the sampled parameters λ_i, $i \in \{2,3,4,5,6\}$, and σ , the daily doses in age group i, and the rate of receiving the second dose, respectively, on the cumulative cases and deaths. We observe that the age groups 3 and 4, namely, 20-39 and 40-59 years present the highest PRCC among the daily doses, suggesting that an increased vaccine

coverage of these age groups leads to the largest reduction in cases and deaths. Moreover, σ is negatively correlated to cases and deaths, suggesting that if this rate is small, hence the time between doses is longer, cases and deaths will increase. Similar results are visible for the hospitalizations reported 50 days after reopening in June.

Table SI12 shows the PRCC of some of the infection-related parameters on the model outcomes. Increase of contact, susceptibility of adults aged between 20 and 59 years show a significant positive correlation on deaths and cases, suggesting that reopening stages and higher susceptibility of adults will generate an increase of the infection,

Table SI12: PRCC on cumulative cases and deaths, investigating infection-related parameters.

**SENSITIVITY ANALYSIS**			
**PARAMETERS**	**DEFINITION**	**PRCC**	
		CASES	DEATHS
c_{inc}	Percentage increase of contacts	**0.6878**	**0.6878**
ϕ_1	Susceptibility age group 0-9	0.4791	0.4791
ϕ_2	Susceptibility age group 10-19	0.4946	0.4946
ϕ_3	Susceptibility age group 20-39	**0.9539**	**0.9539**
ϕ_4	Susceptibility age group 40-59	**0.9158**	**0.9158**
ϕ_5	Susceptibility age group 60-79	0.3994	0.3994
ϕ_6	Susceptibility age group 80+	0.106	0.106

Cases data until December 2021

Figure SI9: Daily cases reported in Toronto from December 2020 to December 2021[15] (green). The dashed lined represents the end of the period of time used to calibrate the model. The curves represent the model outcomes, with highest coverage among adults, under the following scenarios: total reopening in August with second dose given 21 days after first dose; total reopening in September with second dose given 112 days after first dose and lower efficacy; total reopening in September with second dose given 21 days after first dose; partial reopening in September with second dose given 112 days after first dose.

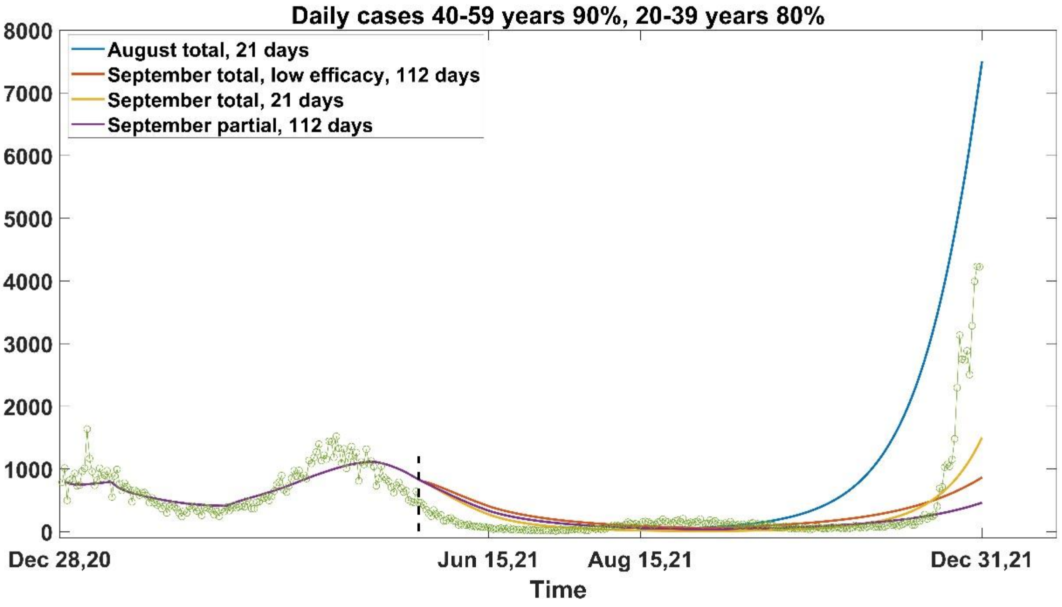

Data following the period of time used for the model calibration show similar trend to our model prediction, with a decrease trend until August 15, followed by a slight increase. towards the end of 2021, we observe a sharp increase, attributable to the emergence of Omicron.

References

[1] Toronto Public Health, COVID-19: Vaccine Data, https://www.toronto.ca/home/covid-19/covid-19-latest-city-of-toronto-news/covid-19-pandemic-data/covid-19-vaccine-data/

[2] Goldstein E, Lipsitch M, Cevik M. On the effect of age on the transmission of SARS-CoV-2 in households, schools and the community. medRxiv [Preprint]. 2020 Jul 24:2020.07.19.20157362. doi: 10.1101/2020.07.19.20157362. Update in: J Infect Dis. 2021 Feb 13;223(3):362-369. PMID: 32743609; PMCID: PMC7386533.

[3] Drolet, M., Lemieux-Mellouki, P., Bureau, A., Mondor M., Hardy M., Gingras G., Brisson, M. CANADIAN MIXING MATRICES DURING THE PRE-PANDEMIC PERIOD AND THE 2020 SPRING LOCKDOWN: DATA FROM CONNECT , https://www.inspq.qc.ca/covid-19/donnees/connect

[4] CDC, About Variants, https://www.cdc.gov/coronavirus/2019-ncov/transmission/variant.html

[5] WHO https://www.who.int/csr/don/archive/disease/novel_coronavirus/en/

[6] Public Health Ontario, COVID-19 B.1.1.7 (501Y.V1) Variant of Concern – What We Know So Far, https://www.publichealthontario.ca/-/media/documents/ncov/covid-wwksf/2020/12/what-we-know-uk-variant.pdf?la=e

[7] NCCID, Updates on COVID-19 Variants of Concern (VOC), https://nccid.ca/covid-19-variants/

[8] Li Q, Guan X, Wu P, et al. Early transmission dynamics in Wuhan,China, of novel coronavirus-infected pneumonia. N Engl J Med 2020; 382: 1199–207.
[9] Li R, Pei S, Chen B, et al. Substantial undocumented infection facilitates the rapid dissemination of novel coronavirus (SARS-CoV2). Science 2020;

[10] Buitrago-Garcia D, Egli-Gany D, Counotte MJ, Hossmann S, Imeri H, Ipekci AM, et al. (2020) Occurrence and transmission potential of asymptomatic and presymptomatic SARS-CoV-2 infections: A living systematic review and meta-analysis. PLoS Med 17(9): e1003346. https://doi.org/10.1371/journal.pmed.1003346

[11] Cevik M, Tate M, Lloyd O, Maraolo AE, Schafers J, Ho A. SARS-CoV-2, SARS-CoV, and MERS-CoV viral load dynamics, duration of viral shedding, and infectiousness: a systematic review and meta-analysis. Lancet Microbe. 2021 Jan;2(1):e13-e22. doi: 10.1016/S2666-5247(20)30172-5. Epub 2020 Nov 19. PMID: 33521734; PMCID: PMC7837230.

[12] Public Health Ontario, COVID-19 Real-World Vaccine Effectiveness – What We Know So Far, https://www.publichealthontario.ca/-/media/documents/ncov/covid-wwksf/2021/04/wwksf-vaccine-effectiveness.pdf?la=en

[13] Ministry of Health Memorandum to: Medical Officers of Health Hospital CEOs https://www.wrh.on.ca/uploads/Coronavirus/MOH_Vaccine_Memo_March_8_2021.pdf

[14] MATLAB fmincon https://www.mathworks.com/help/optim/ug/fmincon.html

[15] City of Toronto, COVID-19: Pandemic Data, https://www.toronto.ca/home/covid-19/covid-19-pandemic-data/
